# Mitochondrial Respiration Is Reduced in Atherosclerosis, Promoting Necrotic Core Formation and Reducing Relative Fibrous Cap Thickness

**DOI:** 10.1161/ATVBAHA.117.310042

**Published:** 2017-09-28

**Authors:** Emma P.K. Yu, Johannes Reinhold, Haixiang Yu, Lakshi Starks, Anna K. Uryga, Kirsty Foote, Alison Finigan, Nichola Figg, Yuh-Fen Pung, Angela Logan, Michael P. Murphy, Martin Bennett

**Affiliations:** From the Division of Cardiovascular Medicine, Addenbrooke’s Centre for Clinical Investigation, Addenbrooke’s Hospital, University of Cambridge, United Kingdom (E.P.K.Y., J.R., H.Y., L.S., A.K.U., K.F., A.F., N.F., M.B.); Department of Biomedical Sciences, University of Nottingham, Malaysia Campus, Selangor, Malaysia (Y.-F.P.); and MRC Mitochondrial Biology Unit, Cambridge, United Kingdom (A.L., M.P.M.).

**Keywords:** atherosclerosis, mitochondria, reactive oxygen species, respiration, vascular smooth muscle

## Abstract

**Objective—:**

Mitochondrial DNA (mtDNA) damage is present in murine and human atherosclerotic plaques. However, whether endogenous levels of mtDNA damage are sufficient to cause mitochondrial dysfunction and whether decreasing mtDNA damage and improving mitochondrial respiration affects plaque burden or composition are unclear. We examined mitochondrial respiration in human atherosclerotic plaques and whether augmenting mitochondrial respiration affects atherogenesis.

**Approach and Results—:**

Human atherosclerotic plaques showed marked mitochondrial dysfunction, manifested as reduced mtDNA copy number and oxygen consumption rate in fibrous cap and core regions. Vascular smooth muscle cells derived from plaques showed impaired mitochondrial respiration, reduced complex I expression, and increased mitophagy, which was induced by oxidized low-density lipoprotein. Apolipoprotein E–deficient (ApoE^−/−^) mice showed decreased mtDNA integrity and mitochondrial respiration, associated with increased mitochondrial reactive oxygen species. To determine whether alleviating mtDNA damage and increasing mitochondrial respiration affects atherogenesis, we studied ApoE^−/−^ mice overexpressing the mitochondrial helicase Twinkle (Tw^+^/ApoE^−/−^). Tw^+^/ApoE^−/−^ mice showed increased mtDNA integrity, copy number, respiratory complex abundance, and respiration. Tw^+^/ApoE^−/−^ mice had decreased necrotic core and increased fibrous cap areas, and Tw^+^/ApoE^−/−^ bone marrow transplantation also reduced core areas. Twinkle increased vascular smooth muscle cell mtDNA integrity and respiration. Twinkle also promoted vascular smooth muscle cell proliferation and protected both vascular smooth muscle cells and macrophages from oxidative stress–induced apoptosis.

**Conclusions—:**

Endogenous mtDNA damage in mouse and human atherosclerosis is associated with significantly reduced mitochondrial respiration. Reducing mtDNA damage and increasing mitochondrial respiration decrease necrotic core and increase fibrous cap areas independently of changes in reactive oxygen species and may be a promising therapeutic strategy in atherosclerosis.

## Abstract 2

Supplemental Digital Content is available in the text.

Mitochondria are cellular powerhouses, fuelling metabolic processes through their generation of ATP. Mitochondria are thus critical for cellular function, yet they hold a degree of independence, with their own genome and time-scale of replication. In addition, mitochondria have pivotal roles in the regulation of cell death, metabolism, and generation of reactive oxygen species (ROS). Damage to mitochondria can, therefore, impair cellular function, potentially promoting aging and disease.

**See accompanying editorial on page 2229**

Mitochondrial DNA (mtDNA) exists as a 16 569 bp circular molecule associated with the mitochondrial inner membrane. mtDNA contains 37 genes, encoding subunits of respiratory complexes I, III, and IV and the ATP synthase complex V, together with ribosomal and transfer RNAs.^1,2^ The respiratory complexes are central to oxidative phosphorylation, where electron transfer to oxygen is coupled with ATP production. However, ROS are formed as a byproduct of the respiratory chain making mitochondria a major source of cellular ROS.^3^ mtDNA is especially vulnerable to damage, partly because mtDNA lies close to the site of ROS production, and also because mtDNA lacks protective histones. mtDNA defects, such as mutations and deletions, can also be introduced through replication error.^4^

mtDNA is replicated by the mtDNA replisome, which comprises the Twinkle helicase, the mtDNA polymerase, and mitochondrial single-stranded DNA-binding protein. Twinkle unwinds short stretches of double-stranded DNA in the 5′-3′ direction and is an important regulator of mtDNA copy number.^5^ Mice expressing mutated Twinkle show multiple mtDNA deletions and develop chronic late-onset mitochondrial disease^6^; in contrast, transgenic mice expressing increased Twinkle levels (Tw^+^) show increased mtDNA copy number, potentially increasing mitochondrial function.^5^

mtDNA damage can result in mitochondrial dysfunction, leading to the proatherogenic processes of inflammation and apoptosis. Indeed, mtDNA damage is present in the aortas, hearts, and circulating leukocytes of patients with atherosclerosis^7–9^ and is an early event in atherogenesis in apolipoprotein E–deficient (ApoE^−/−^) mice.^8^ Furthermore, large-scale induction of mtDNA damage can directly promote atherosclerosis and plaque vulnerability in ApoE^−/−^ mice independent of ROS.^9^ However, it is not clear what causes mtDNA damage in atherosclerosis and importantly whether the endogenous levels of mtDNA damage seen in mouse or human atherosclerosis are sufficient to cause mitochondrial dysfunction. It is also unclear whether alleviating mtDNA damage and improving mitochondrial respiration affect plaque burden or composition and whether any effects are independent of changes in ROS or metabolism.

We find that endogenous levels of mtDNA damage in mouse and human atherosclerosis are sufficient to reduce mitochondrial copy number and respiration. In contrast, increasing mtDNA integrity and copy number through overexpression of Twinkle increases mitochondrial respiration independent of changes in ROS, promotes vascular smooth muscle cell (VSMC) proliferation, and reduces VSMC and macrophage apoptosis. This results in decreased necrotic core and increased fibrous cap areas.

## Materials and Methods

Materials and Methods are available in the online-only Data Supplement.

## Results

### Mitochondrial Dysfunction Is Present in Human Atherosclerosis

**Figure 1.**
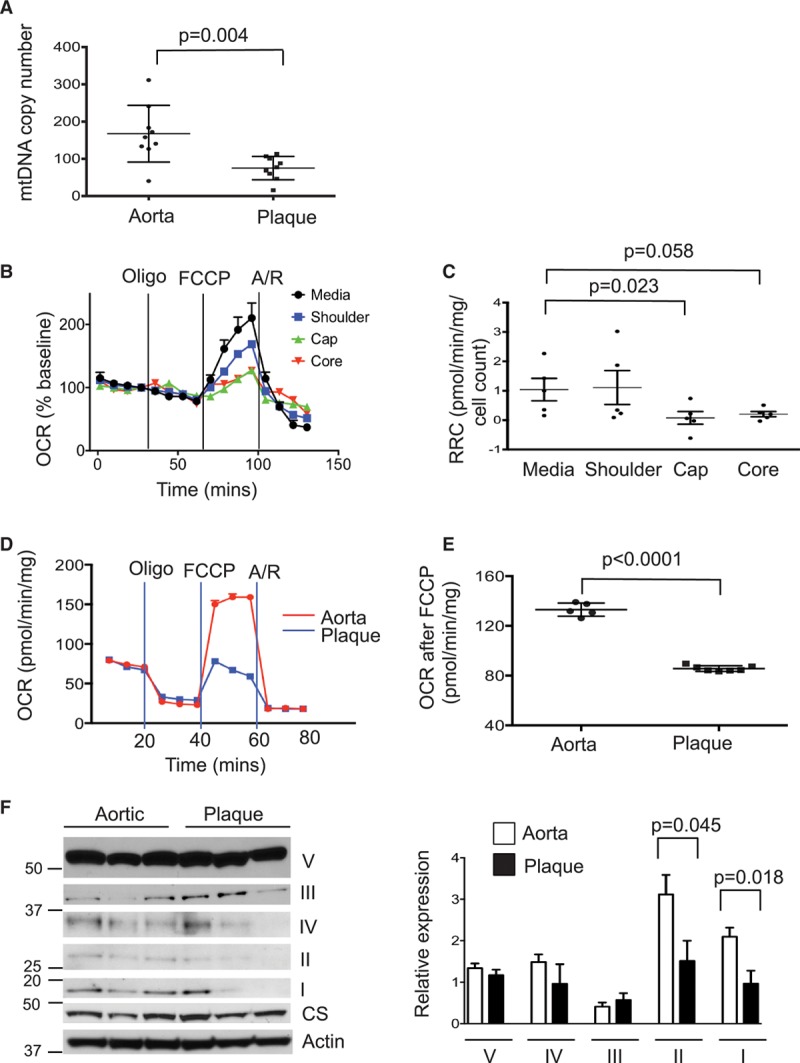
Human atherosclerosis shows reduced mitochondrial copy number and respiration. **A**, Mitochondrial copy number in normal human aorta and plaque (n=9). **B** and **C**, Seahorse profile for oxygen consumption rate (OCR) in human plaque segments from the media, shoulder, cap, and core with treatment with oligomycin (oligo), carbonyl cyanide-4-(trifluoromethoxy)phenylhydrazone (FCCP), and antimycin/rotenone (A/R; **B**) and respiratory reserve capacity (RRC; **C**; n=5–6). Representative Seahorse profile of human plaque vascular smooth muscle cells (VSMCs; blue) and normal aortic VSMCs (red; **D**) and OCR after FCCP (**E**) from n=5 to 7 cultures. **F**, Western blot and quantification of protein expression of mitochondrial protein complexes within the electron transport chain (complexes I–V) in normal human aortic or plaque VSMCs (n=3–5) relative to citrate synthase (CS). Data are mean±SEM. mtDNA indicates mitochondrial DNA.

To determine whether mtDNA damage in human plaques results in functional consequences, such as reduced mtDNA copy number or mitochondrial respiration, human plaques were obtained from carotid endarterectomies and normal (undiseased) aortas from patients undergoing aortic valve surgery. Patient age and sex were similar in both groups (n=9 per group; mean age, 71.8±8.3 and 70.6±11.1 years for plaques and aortas, respectively, and 66.7% male for both groups). Human plaques had significantly reduced mtDNA copy number compared with normal aorta (Figure 1A); this was not because of reduced cell number because copy number was controlled against nuclear DNA. Mitochondrial respiration was determined using a Seahorse extracellular flux analyzer to assess mitochondrial oxygen consumption rate (OCR) and the respiratory reserve capacity, which assesses the potential to increase respiration after uncoupling electron transport. Plaques were microdissected into regions containing the media, the shoulder region, the fibrous cap, and the necrotic core (Figure I in the online-only Data Supplement) and normalized for wet weight. Cell nuclei were subsequently counted in each tissue segment to correct for differences in cellularity. OCR was similar in the media and plaque regions at baseline, but the cap (*P*=0.019) and core (*P*=0.024) regions showed much lower increases after carbonyl cyanide-4-(trifluoromethoxy)phenylhydrazone (FCCP) uncoupling (Figure 1B). Similarly, respiratory reserve capacity was markedly reduced in cap versus media with a similar trend in core versus media (Figure 1C).

### Plaque VSMCs Show Markedly Reduced Mitochondrial Respiration

Plaques comprise several different cell types, and thus whole plaque assays do not identify which cells have reduced mitochondrial respiration. We therefore cultured VSMCs from plaques and normal vessels and analyzed their mitochondrial respiration by Seahorse. VSMCs derived from these endarterectomies are from the intima and part of the underlying media, and aortic VSMCs were from the media. OCR was similar in plaque versus normal aortic VSMCs at baseline but significantly reduced after uncoupling (Figure 1D and 1E). Western blot of the mitochondrial protein complexes within the electron transport chain (complexes I–V) showed significant heterogeneity of expression both within and between cell lines; however, complexes I and II expression were significantly reduced in cultured plaque versus normal aortic VSMCs normalized to citrate synthase (Figure 1F).

### Mitophagy Is Increased by Oxidized Low-Density Lipoprotein and in Plaque VSMCs

**Figure 2.**
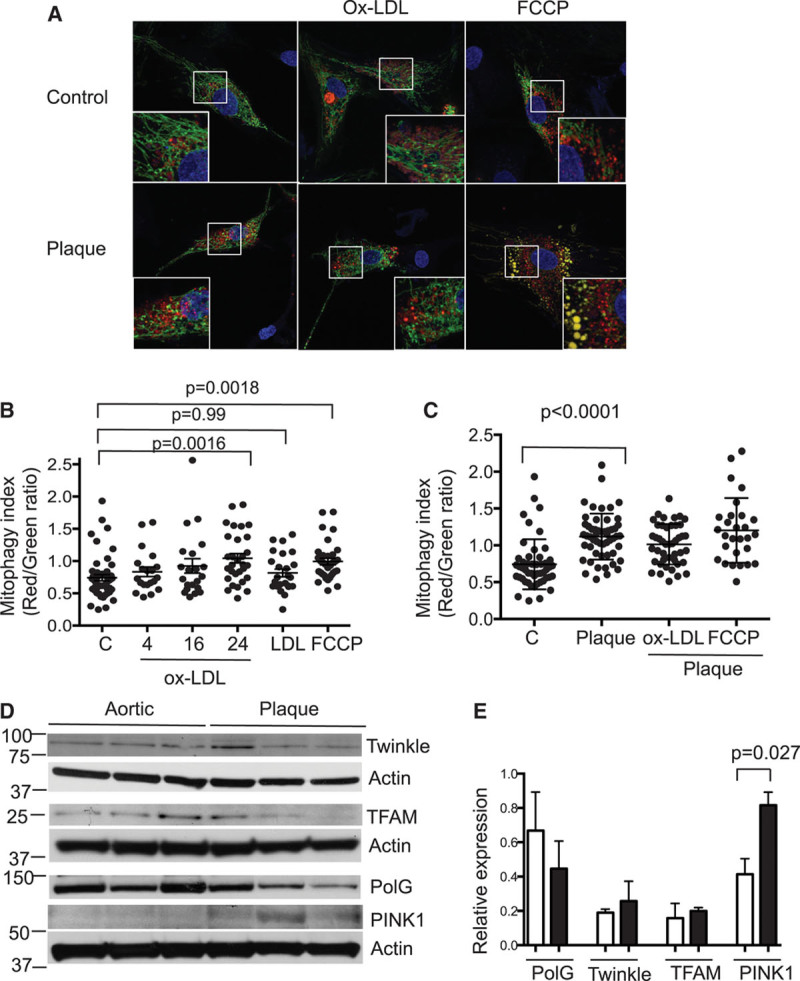
Mitophagy is increased by oxidized low-density lipoprotein (ox-LDL) and in plaque vascular smooth muscle cells (VSMCs). **A**, Confocal microscopic images of control normal human aortic or plaque VSMCs expressing mitochondrially targeted keima, either untreated or after treatment with 100 mg/mL ox-LDL or 5 mmol/L carbonyl cyanide-4-(trifluoromethoxy)phenylhydrazone (FCCP). Insets show high-power views of outlined areas. **B** and **C**, Red/green keima ratios for individual normal aortic VSMCs either untreated (**C**) or treated with 100 mg/mL ox-LDL for 4 to 24 h, 100 mg/mL native LDL for 24 h, or 5 mmol/L FCCP (**B**), or individual normal aortic VSMCs (**C**) or human plaque VSMCs either untreated or treated with ox-LDL or FCCP for 24 h (**C**). n≥20 images from 3 biological replicates. Western blots (**D**) and their quantification (**E**) for proteins involved in mitochondrial DNA synthesis and mitophagy in human aortic or plaque VSMCs, normalized to actin (n=3). PINK1 indicates PTEN-induced putative kinase 1; PolG, polymerase-γ; and TFAM, mitochondrial transcription factor A.

Mitochondrial mass is determined by the balance between mitochondriogenesis and mitochondrial autophagy (mitophagy), both of which are highly regulated processes.^10^ Autophagy has been shown previously to protect against atherosclerosis^11^ while oxidized low-density lipoprotein (ox-LDL) can induce mitophagy in VSMCs.^12^ Mitophagy was determined by infecting human VSMCs with a lentivirus encoding a mitochondrially targeted keima protein, which fluoresces with peak emission at 620 nm and a pH-dependent excitation maximum of 440 nm at pH 7 to 8, the normal pH of the mitochondrial matrix (coded green), or 586 nm at pH 4 to 5, the pH of lysosomes targeting mitochondria for mitophagy (coded red).^13^ Because keima is resistant to lysosomal degradation, a ratio of red/green keima fluorescence can estimate mitophagy.^14^ Ox-LDL but not native LDL induced time-dependent mitophagy, which was also seen after FCCP (Figure 2A and 2B). Human plaque VSMCs also showed increased mitophagy compared with normal VSMCs, which was not further increased by ox-LDL or FCCP (Figure 2C).

mtDNA synthesis is regulated by several genes, including Twinkle, mtDNA polymerase-γ, and mitochondrial transcription factor A (TFAM). Expressions of Twinkle, polymerase-γ, and TFAM were similar in aortic and plaque VSMCs (Figure 2D and 2E). In contrast, mitophagy is regulated, in part, by the expression and localization of the serine-threonine kinase phosphatase and tensin homolog (PTEN)–induced putative kinase 1 (PINK1) and its interaction with the ubiquitin ligase parkin. PINK1 is normally expressed at low levels but is stabilized and becomes detectable during mitophagy. PINK1 expression was significantly increased in plaque versus normal VSMCs, consistent with increased mitophagy (Figure 2D and 2E). Together, these findings suggest that plaque VSMCs undergo increased mitophagy, most likely induced by ox-LDL, which is not compensated by increased mitochondriogenesis.

### mtDNA Damage and Dysfunction Are Present in Mouse Atherosclerosis

**Figure 3.**
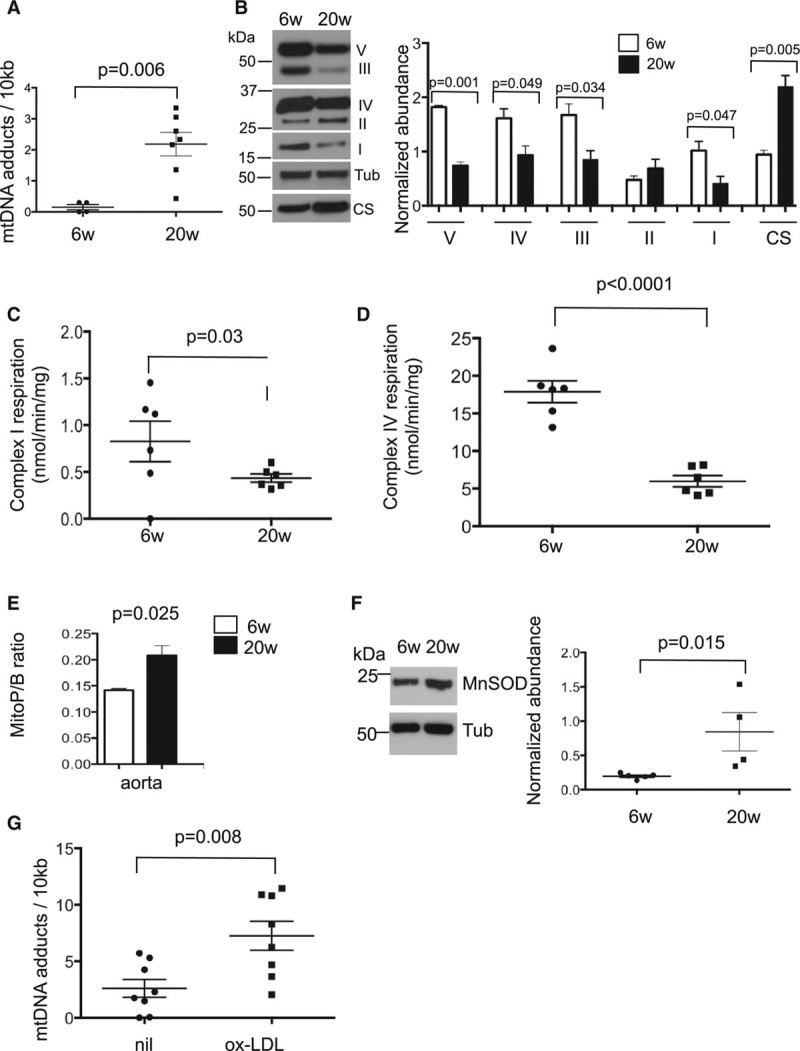
Mitochondrial DNA (MtDNA) damage reduced mitochondrial respiration and increased ROS accumulation during murine atherosclerosis development. Six-wk-old chow-fed apolipoprotein E–deficient (ApoE^−/−^) mice were compared with ApoE^−/−^ mice fat fed from 6 to 20 wk (n=4–7) and aortas examined for (**A**) mtDNA adducts and (**B**) mitochondrial complex or citrate synthase (CS) abundance. Representative Western blot (**left**) and quantification (**right**). Complex I- (**C**) and IV-supported respiration (**D**). **E**, Reactive oxygen species as measured by MitoP/B ratio in aortas. **F**, Representative Western blot (**left**) and quantification (**right**) of aortic manganese superoxide dismutase (MnSOD) abundance at 6 and 20 wk. **G**, mtDNA adducts in ApoE^−/−^ vascular smooth muscle cells isolated from 12- to 16-week-old mice with or without treatment with 100 μg/mL oxidized low-density lipoprotein (ox-LDL) for 24 h (n=8 biological replicates). Tub indicates tubulin.

mtDNA damage is found in ApoE^−/−^ mouse aortas^8,9^; however, whether the mtDNA damage is sufficient to affect mitochondrial function and the cause of endogenous mtDNA damage are unclear. mtDNA damage was assessed by quantitative polymerase chain reaction for mtDNA adducts which halt polymerase progression; damage can therefore be quantified by comparing the amplification of a large mtDNA product to a short target to control for copy number.^8^ mtDNA damage was increased in ApoE^−/−^ mice fat fed for 14 weeks compared with 6-week-old control mice (Figure 3A), associated with reduced abundance of the mitochondrial respiratory complexes that have mtDNA-encoded subunits (complexes I, III, IV, and V) but not citrate synthase (Figure 3B). Respirometry on permeabilized mouse aortas also showed that fat-fed ApoE^−/−^ mice had reduced complex I- and complex IV-supported aortic respiration (Figure 3C and 3D).

mtDNA lies close to the site of ROS production, such that ROS may induce the mtDNA damage associated with atherosclerosis; however, assessment of mitochondrial ROS during atherogenesis has not been reported. Mitochondrial H_2_O_2_ was assessed in vivo in ApoE^−/−^ mice using the mitochondria-targeted probe MitoB. MitoB forms MitoP on reaction with mitochondrial H_2_O_2_ (and also with peroxynitrite), changes that can be assessed by quantifying the MitoP/MitoB ratio using mass spectroscopy.^15^ MitoP/MitoB was increased in the aortas of fat-fed ApoE^−/−^ mice (Figure 3E) and was associated with increased abundance of the antioxidant enzyme manganese superoxide dismutase (Figure 3F). Similar to the whole mouse with fat feeding, ox-LDL treatment of mouse VSMCs in culture induced marked mtDNA damage (Figure 3G).

### Tw^+^/ApoE^−/−^ Mice Show Increased mtDNA Integrity, Copy Number, and Mitochondrial Respiration With No Change in ROS

Our data indicate that atherosclerosis is associated with reduced mitochondrial respiration in human and mouse atherosclerosis. We, therefore, used Twinkle transgenic mice (Tw^+^) to determine whether alleviating mtDNA damage and increasing mitochondrial respiration could affect atherogenesis or plaque composition. Tw^+^ mice express mouse Twinkle helicase intron 4 cDNA under the control of the human β actin promoter, which results in widespread transgene expression, including in muscle-containing tissues.^5^ Tw^+^ mice show increased mtDNA copy number that varies between organs^5^ and reduced mtDNA damage after oxidative stress.^16^ We crossed C57Bl/6 Tw^+^ mice with ApoE^−/−^ mice and examined Twinkle expression, copy number, mtDNA damage, and mitochondrial respiration in aortas and cultured cells. Tw^+^/ApoE^−/−^ mice had increased Twinkle mRNA in aortas, aortic VSMCs, and bone marrow–derived macrophages compared with ApoE^−/−^ controls although the extent of Twinkle expression varied between tissues (Figure II in the online-only Data Supplement). mtDNA copy number was not increased in Tw^+^/ApoE^−/−^ aortas or VSMCs, but mtDNA damage was significantly decreased in both (Figure III in the online-only Data Supplement). Conversely, Tw^+^/ApoE^−/−^ macrophages showed increased mtDNA copy number but no change in mtDNA damage compared with ApoE^−/−^ controls (Figure III in the online-only Data Supplement).

**Figure 4.**
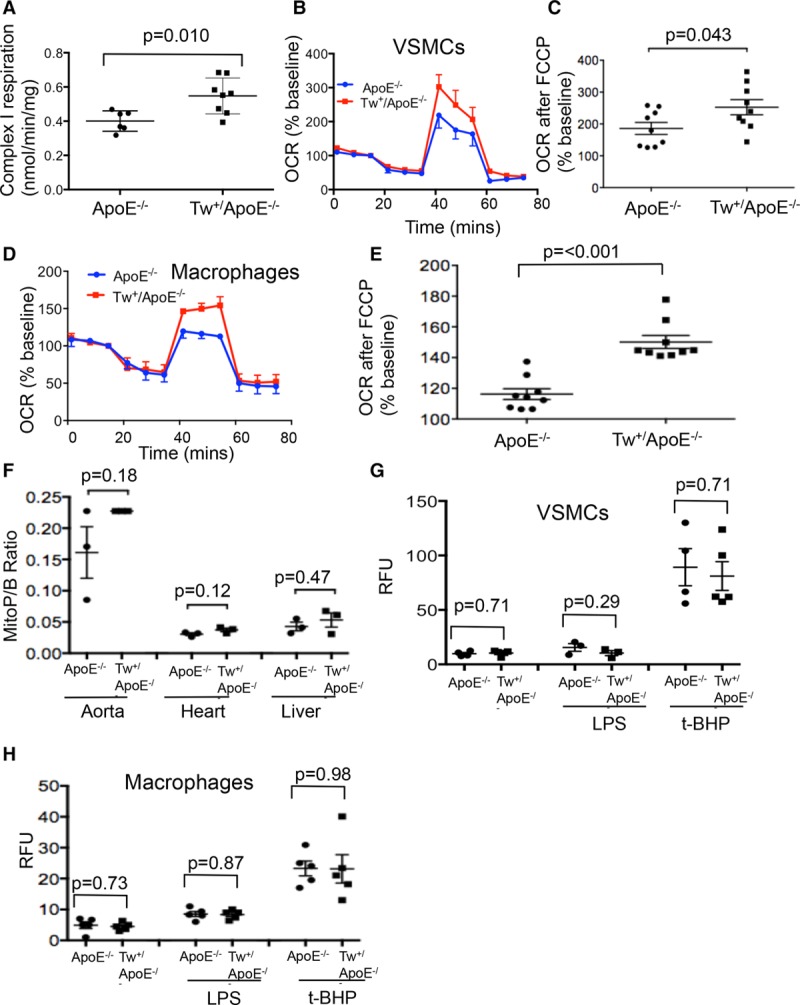
Twinkle increases mitochondrial respiration in aorta, vascular smooth muscle cells (VSMCs), and macrophages. **A**, Ex vivo respirometry for complex I-supported respiration in aortas from control apolipoprotein E–deficient (ApoE^−/−^) or Tw^+^/ApoE^−/−^ mice (n=6–8) after 14-wk high-fat diet (HFD). Representative Seahorse profiles for oxygen consumption rate (OCR; **B** and **D**) or OCR after cyanide-4-(trifluoromethoxy)phenylhydrazone (FCCP; **C** and **E**) in VSMCs (**B** and **C**) or macrophages (**D** and **E**) from control ApoE^−/−^ or Tw^+^/ApoE^−/−^ mice (n=6–9). After 14-wk HFD, reactive oxygen species were measured by MitoP/B ratio in aortas, hearts, and livers (**F**) or dichlorodihydrofluorescein diacetate (DCFDA) relative fluorescence units (RFU) in VSMCs (**G**) or macrophages (**H**) from control ApoE^−/−^ or Tw^+^/ApoE^−/−^ mice. Cultured VSMCs and macrophages were also treated with lipopolysaccharide (LPS) or tert-butyl hydroperoxide (t-BHP). n=4 to 5. Tw indicates Twinkle helicase.

To determine the functional consequences of increased mtDNA copy number or reduced mtDNA damage, we assessed expression of mitochondrial respiratory complexes and respiratory function in Tw^+^/ApoE^−/−^ and ApoE^−/−^ control mice. Tw^+^/ApoE^−/−^ aortas showed increased complex I abundance, and Tw^+^/ApoE^−/−^ macrophages showed increased abundance of both complexes I and III, whereas cultured VSMCs showed no significant difference in complex expression between groups (Figure IV in the online-only Data Supplement). In contrast, Tw^+^/ApoE^−/−^ aortas showed increased complex I-supported respiration (Figure 4A) but no differences in complex II- or IV-supported respiration (Figure V in the online-only Data Supplement), and both Tw^+^/ApoE^−/−^ VSMCs (Figure 4B and 4C) and macrophages (Figure 4D and 4E) had increased OCR after uncoupling with FCCP compared with ApoE^−/−^ controls.

Although, mitochondrial ROS were increased in aortas as atherosclerosis develops (Figure 3E), mtDNA damage can promote atherosclerosis independent of changes in ROS.^9^ We, therefore, assayed ROS in both tissues and cells of ApoE^−/−^ and Tw^+^/ApoE^−/−^ mice. There were no significant differences in the MitoP/MitoB ratio in a range of tissues between control ApoE^−/−^ and Tw^+^/ApoE^−/−^ mice after 14 weeks of high-fat diet (Figure 4F). Similarly, ROS levels in control ApoE^−/−^ or Tw^+^/ApoE^−/−^ VSMCs or macrophages determined using the ROS-sensitive dye dichlorodihydrofluorescein diacetate were similar at baseline and after stimulation with lipopolysaccharide or the free radical-generating agent tert-butyl hydroperoxide (Figure 4G and 4H), indicating that Tw overexpression does not affect ROS levels in vitro or in vivo.

### Tw^+^/ApoE^−/−^ Mice Show Increased Fibrous Cap and Decreased Necrotic Core Areas

**Figure 5.**
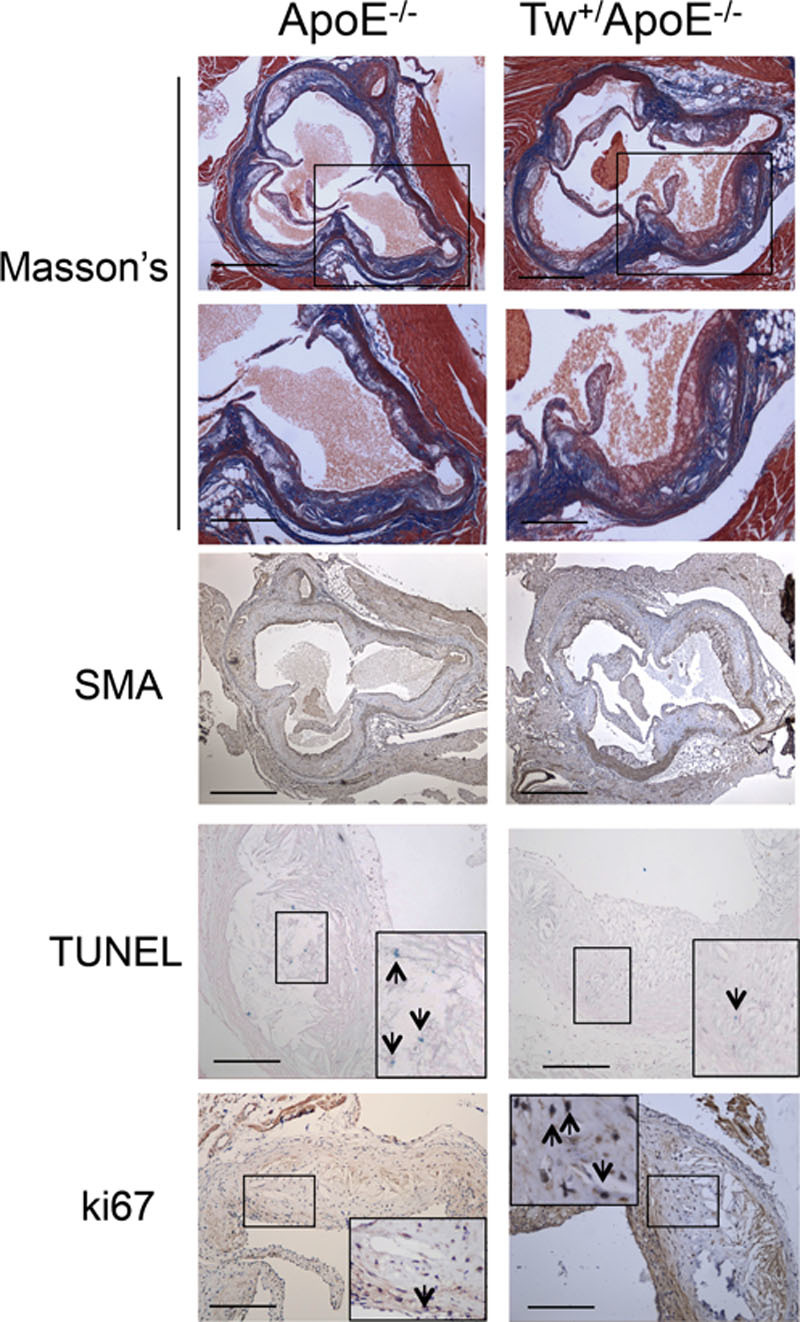
Twinkle mice show reduced necrotic core and increased fibrous cap areas. Histochemistry and immunohistochemistry of aortic root plaques of control apolipoprotein E–deficient (ApoE^−/−^) and Tw^+^/ApoE^−/−^ mice after 14 wk of fat feeding. Sections were stained with Masson’s trichrome or antibodies to α-smooth muscle actin (α-SMA) or ki67 or underwent TUNEL. Scale bar: low power, 500 μm; high power, 100 μm. Tw indicates Twinkle helicase.

**Figure 6.**
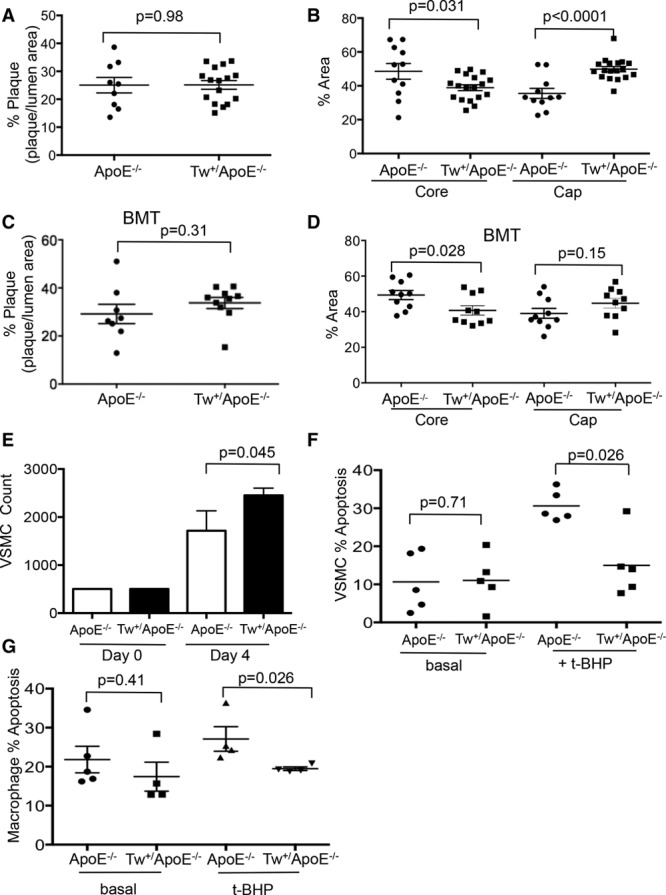
Twinkle mouse plaques show reduced necrotic core and increased fibrous cap areas. Aortic root plaque % (**A**), necrotic core, and fibrous cap % areas (**B**) in control apolipoprotein E–deficient (ApoE^−/−^) and Tw^+^/ApoE^−/−^ mice after fat feeding from 6 to 20 wk (n=11–18). Aortic root plaque % (**C**), necrotic core, and fibrous cap % areas (**D**) in ApoE^−/−^ mice transplanted with control ApoE^−/−^ or Tw^+^/ApoE^−/−^ bone marrow at 6 wk and fat feeding from 6 to 20 w (n=8–10). **E**, Cell number at day 0 and day 4 in vascular smooth muscle cells (VSMCs) derived from control ApoE^−/−^ and Tw^+^/ApoE^−/−^ mice (n=3). Apoptosis at baseline and after 50 μmol/L tert-butyl hydroperoxide (t-BHP) for 16 h in VSMCs (**F**) and macrophages (**G**) from control ApoE^−/−^ and Tw^+^/ApoE^−/−^ mice (n=4–5). BMT indicates bone marrow transplant; and Tw, Twinkle helicase.

To determine whether improved mitochondrial respiration could affect atherosclerosis, male and female Tw^+^/ApoE^−/−^ and ApoE^−/−^ littermate controls were fat fed from 6 to 20 weeks of age. There were no differences in body weight, blood pressure, blood counts, hemoglobin, serum lipids, or glucose between groups (Figure VI in the online-only Data Supplement). Detailed metabolic phenotyping using comprehensive laboratory animal monitoring system cages also showed no differences in food/water consumption, activity (as assessed by beam breaks), and total oxygen consumption (Figure VII in the online-only Data Supplement). Atherosclerosis was examined in aortic roots, brachiocephalic arteries, and the descending aorta. There was no difference in plaque burden between Tw^+^/ApoE^−/−^ and ApoE^−/−^ mice in any vascular bed (Table; Figures 5 and 6A). However, Twinkle overexpression affected plaque composition, with Tw^+^/ApoE^−/−^ plaques showing increased fibrous cap and decreased necrotic core areas (Table; Figures 5 and 6B). Tw^+^/ApoE^−/−^ plaques also showed a borderline increase in smooth muscle actin–positive area, significantly increased proliferating cells, and significantly decreased cells undergoing apoptosis (Table; Figure 5).

**Table.**
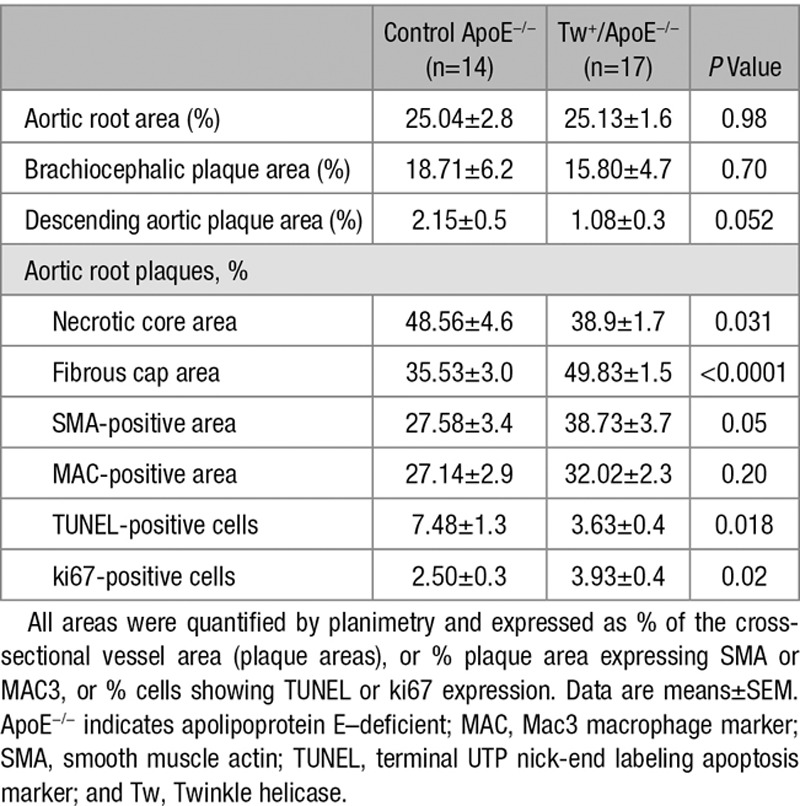
Analysis of Atherosclerotic Plaques From Control ApoE−/− and Tw+/ApoE−/− Mice

To distinguish between the effects of Twinkle overexpression on vessel wall or bone marrow–derived cells, 6-week-old ApoE^−/−^ mice were irradiated and transplanted with Tw^+^/ApoE^−/−^ or control ApoE^−/−^ bone marrow, fat fed from 6 to 20 weeks, and atherosclerosis examined. There was no difference in body weight, blood counts, hemoglobin or serum lipids, or glucose between mice receiving Tw^+^/ApoE^−/−^ or control ApoE^−/−^ bone marrow (Figure VI in the online-only Data Supplement). Twinkle expression and complex I-, II-, or IV-supported respiration were similar in the aortas from mice transplanted with control or Tw^+^ marrow (Figure VIII in the online-only Data Supplement). Although there was no difference in the extent of atherosclerosis (Figure 6C), Tw^+^ marrow transplantation decreased the necrotic core area while fibrous cap areas were similar between groups (Figure 6D).

### Effects of Twinkle Overexpression on VSMCs and Macrophages

The presence of increased fibrous cap and decreased necrotic core areas in Tw^+^/ApoE^−/−^ mice, together with increased numbers of proliferating cells and reduced numbers of apoptotic cells, suggests that Twinkle overexpression may have direct effects on cells in the atherosclerotic plaque. Indeed, Tw^+^/ApoE^−/−^ VSMCs showed increased cell counts after 4 days in culture (Figure 6E), and because there was no difference in baseline apoptosis (Figure 6F), this indicates increased proliferation compared with control ApoE^−/−^ VSMCs. Tw^+^ VSMCs and macrophages also showed reduced apoptosis after tert-butyl hydroperoxide–induced oxidative stress (Figure 6F and 6G) while Tw^+^/ApoE^−/−^ macrophages showed no difference in the secretion of a range of cytokines (Figure IX in the online-only Data Supplement).

## Discussion

Mitochondria regulate multiple processes that are key to both cellular and organismal health, including ATP and ROS generation, cell death, inflammation, and systemic metabolism. We and others have shown that human and mouse atherosclerotic plaques show mtDNA damage^8,9^ and that extensive, supraphysiological levels of mtDNA damage promote atherosclerosis through effects on apoptosis, inflammation, and systemic metabolism.^9^ However, it is unclear whether the endogenous levels of mtDNA damage observed in mouse and human atherosclerosis are sufficient to reduce mitochondrial respiration, whether improving mitochondrial respiration affects atherogenesis and plaque composition, and what the mechanisms involved are.

Our study demonstrates several important findings. We show for the first time that the mtDNA damage seen in human and mouse atherosclerosis is sufficient to reduce mitochondrial respiration, is most marked in the cap and core regions, and is associated with reduced mtDNA copy number in human plaques. Cultured human plaque VSMCs show reduced mitochondrial respiration and reduced expression of complex I. Ox-LDL induces mtDNA damage and mitophagy in VSMCs, suggesting a possible cause for the mtDNA damage and dysfunction in atherosclerosis, and human plaque VSMCs show increased mitophagy. The Twinkle helicase increases mtDNA copy number and respiratory complex expression, resulting in improved mitochondrial respiration in the whole aorta, VSMCs, and macrophages. Twinkle increases VSMC proliferation and protects VSMCs and macrophages against oxidant stress–induced apoptosis. Importantly, Twinkle overexpression during atherogenesis increases the size of the fibrous cap and reduces necrotic core areas while also increasing cell proliferation and reducing apoptosis in vivo. Reduced necrotic core areas were also seen after transplantation with Tw^+^ marrow, suggesting that effects on both bone marrow–derived cells and VSMCs are important. Our study suggests that preventing the mtDNA damage and dysfunction seen in atherosclerosis may be important in modulating plaque composition.

Mitochondria can sustain extensive damage without affecting overall function, in part because of compensatory mitochondriogenesis. However, we show that the mtDNA damage seen in mouse and human atherosclerosis is sufficient to reduce complex expression, mitochondrial respiration, and reserve capacity, particularly in the cap and core regions in human plaques. OCR and respiratory reserve capacity were corrected for both tissue weight and cell number, and reduced complex I- and IV-supported respirations were also seen in aortas of ApoE^−/−^ mice, indicating that these differences are not just because of cell death. This suggests that the cells comprising the plaque develop progressive mtDNA damage and dysfunction, most likely because of exposure to risk factors, such as aging, diabetes mellitus, hypertension, smoking, and hyperlipidemia. Indeed, we show that ox-LDL can induce mtDNA damage and mitophagy, and human plaque VSMCs showed increased mitophagy. Autophagy is generally considered a protective mechanism, including in human VSMCs induced by atherogenic lipids^12^; however, mitophagy in the absence of compensatory mitochondriogenesis may ultimately reduce mitochondrial respiration.

To augment mitochondrial respiration, we studied atherosclerosis in mice overexpressing the Twinkle helicase, both as global overexpression and after bone marrow transplant. Twinkle overexpression increased copy number in macrophages, similar to previous findings in heart and muscle.^5^ Tw^+^/ApoE^−/−^ aortas and VSMCs showed no change in copy number but did have reduced mtDNA damage. Tissue/cell-specific effects of Twinkle overexpression have been described previously and may reflect differences in mtDNA replication and turnover.^5^ However, improvements in mtDNA integrity or copy number both increased respiratory complex abundance and improved mitochondrial respiration.

The augmented mitochondrial respiration in Tw^+^ mice may also directly affect cellular phenotype. Mitochondria regulate proliferation through multiple pathways, including through aspartate and ATP synthesis; the resulting increased ATP/AMP ratio would lead to decreased AMP-activated protein kinase activation and less cell cycle suppression.^17^ Mitochondria are also important regulators of apoptosis through the mitochondrial permeability transition pore.^2^ Mitochondrial permeability transition pore opening releases death-promoting factors, such as cytochrome c and apoptosis-inducing factor.^18,19^ Improving mitochondrial respiration may lead to increased ATP production, which would reduce pore opening and thus cell death.

Mitochondria are a major source of cellular ROS, but the exact relationship between mtDNA damage, dysfunction, and ROS is unclear. Improving mtDNA integrity might reduce ROS levels, and reduced ROS has been observed previously in Twinkle-overexpressing cells.^20^ In contrast, although Twinkle overexpression improved mitochondrial respiration, we found no changes in ROS in either arteries in vivo or VSMCs or macrophages in vitro. Our study shows that while aortic mitochondrial ROS do increase during murine atherogenesis, augmenting mtDNA integrity and mitochondrial respiration can affect plaque composition independently of changes in ROS.

We find that selectively improving mtDNA copy number, integrity, and respiration increases fibrous cap and reduces necrotic core areas and increases cell proliferation and reduces apoptosis in Tw^+^/ApoE^−/−^ mice. There were no systemic metabolic changes in Tw^+^/ApoE^−/−^ mice, suggesting that the changes in plaque composition are because of changes in vessel wall cells and macrophages. VSMCs provide the protective fibrous cap in established plaques, and their apoptosis and senescence promote cap thinning.^21^ Macrophage apoptosis also increases plaque necrosis.^22^ In contrast, Tw^+^/ApoE^−/−^ VSMCs show increased cell proliferation, and Tw^+^/ApoE^−/−^ VSMCs, macrophages, and plaques show reduced apoptosis. The independent effect of Twinkle on myeloid cells is also demonstrated by the reduction in core size seen after transplantation with Tw^+^ marrow.

Our data support a model where ROS/ox-LDL induce mtDNA damage, which is sufficient to decrease copy number, reduce complex I expression, promote mitophagy without compensatory mitochondriogenesis, and reduce mitochondrial respiration. Mitochondrial dysfunction promotes VSMC and macrophage apoptosis and reduces VSMC proliferation leading to increased necrotic core and decreased fibrous cap areas. However, recovery of mtDNA integrity, for example, by Twinkle overexpression, improves mitochondrial respiration, promotes VSMC proliferation, and protects against VSMC and macrophage apoptosis. Plaque composition is thus altered, with decreased necrotic core and increased fibrous cap areas.

Our study has some limitations. First, although the reduced mtDNA copy number and mitochondrial respiration in human plaques and plaque-derived VSMCs may be because of ox-LDL–mediated mtDNA damage and mitophagy, we were not able to directly examine the cause of reduced mitochondrial respiration in human plaques or examine mitophagy in mouse plaques. Second, we examined respiration in normal aortic versus diseased carotid VSMCs, and cell origin might affect our results. However, our previous studies have shown that DNA damage is similar in normal (undiseased) arteries from different vascular beds.^23^ Third, we studied mouse bone marrow–derived macrophages rather than resident macrophages exposed to the high-fat diet. However, plaque macrophages are mainly derived from bone marrow precursors, although as bone marrow–derived macrophages are cultured ex vivo for 7 days, the extent the cells retain the effects of high-fat feeding is uncertain. Last, the Tw^+^ mice show improved mtDNA integrity, copy number, and respiration over the timeframe under study (up to 20 weeks) and in specific tissues. However, Twinkle overexpression has different effects on different tissues, and we cannot exclude beneficial effects of Twinkle overexpression on other cells in the plaque, such as endothelial cells. In addition, high Twinkle expression can cause respiratory complex deficiency, particularly in tissues with high mitochondrial content.^24^ Twinkle itself is, therefore, unlikely to be a therapeutic target although protecting and augmenting mitochondrial function by pharmacological means may be more successful.

In summary, we show that the endogenous levels of mtDNA damage in mouse and human atherosclerosis are associated with significant reductions in mitochondrial copy number and respiration. Twinkle overexpression improves mtDNA integrity and copy number, resulting in improved mitochondrial respiration and increased fibrous cap and decreased necrotic core areas. Maintaining and restoring mtDNA integrity and mitochondrial function is, therefore, a promising therapeutic strategy.

## Sources of Funding

This work was supported by British Heart Foundation (BHF) grants PG/14/69/31032 and RG/13/14/30314, a Wellcome Trust PhD Fellowship to J. Reinhold, the National Institute for Health Research Cambridge Biomedical Research Centre, the BHF Centre for Research Excellence, the Academy of Medical Sciences and by grants to M.P. Murphy from the Medical Research Council UK (MC_U105663142), and by a Wellcome Trust Investigator award (110159/Z/15/Z).

## Disclosures

None.

## Supplementary Material

## Notes

Highlights

- Mitochondrial respiration is markedly reduced in human atherosclerosis and in vascular smooth muscle cells derived from plaques.
- Mitophagy is increased in plaque vascular smooth muscle cells and after oxidized high-density lipoprotein.
- Augmentation of mitochondrial respiration by overexpression of the Twinkle helicase reduces multiple features of plaque vulnerability.
- Twinkle promotes vascular smooth muscle cell proliferation and reduces apoptosis of vascular smooth muscle cells and macrophages.
- Augmentation of mitochondrial respiration may be a therapeutic target in atherosclerosis.
