# Supplementary figures and images for: Mitochondrial Respiration Is Reduced in Atherosclerosis, Promoting Necrotic Core Formation and Reducing Relative Fibrous Cap Thickness
Source: Arterioscler Thromb Vasc Biol. 2017 Sep 28;37(12):2322–32. doi: 10.1161/ATVBAHA.117.310042 (PMC5701734; doi:10.1161/ATVBAHA.117.310042)

## Graphical Abstract

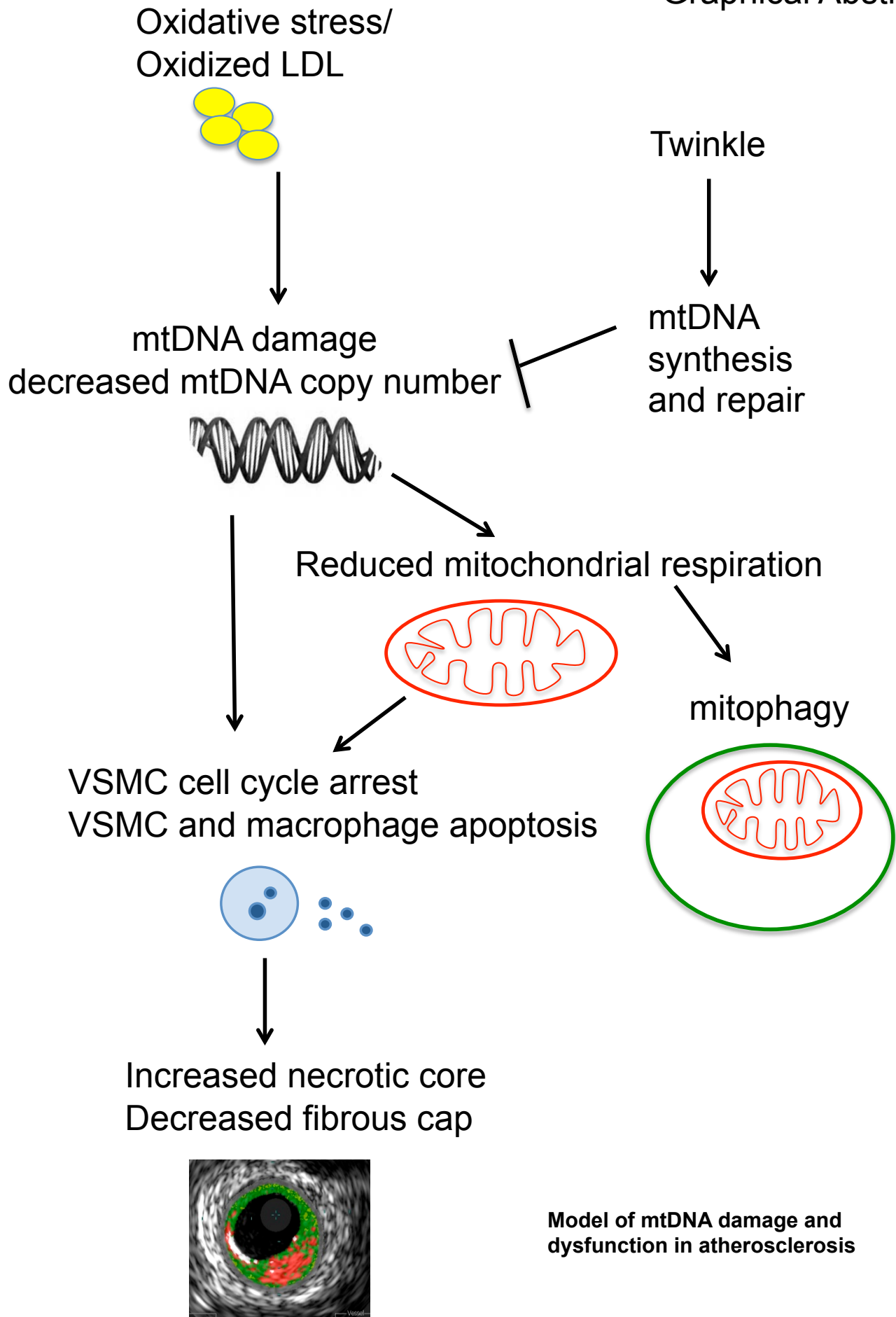

Supplement: Supplementary file 1 [file atv-37-2322-s001.pdf]
