# Supplementary material for: Mitochondrial Respiration Is Reduced in Atherosclerosis, Promoting Necrotic Core Formation and Reducing Relative Fibrous Cap Thickness
Source: Arterioscler Thromb Vasc Biol. 2017 Sep 28;37(12):2322–32. doi: 10.1161/ATVBAHA.117.310042 (PMC5701734; doi:10.1161/ATVBAHA.117.310042)
Supplement: Supplementary file 2 [file atv-37-2322-s002.pdf]

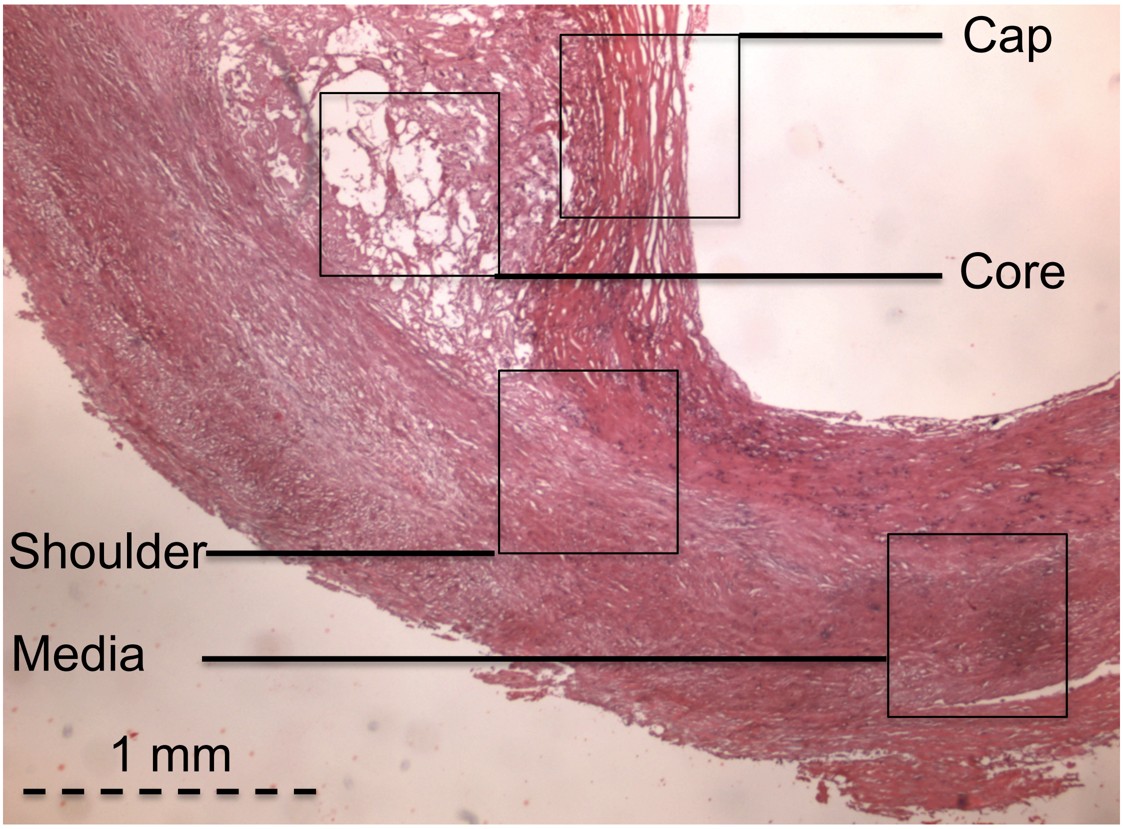

Supplemental Figure I. Illustration of sample areas for Seahorse experiments of carotid endarterectomies.
Hematoxylin and eosin stain of human carotid endarterectomy sample. Top right: lumen. Sample areas used for Seahorse experiments and scale bar are depicted for illustration.

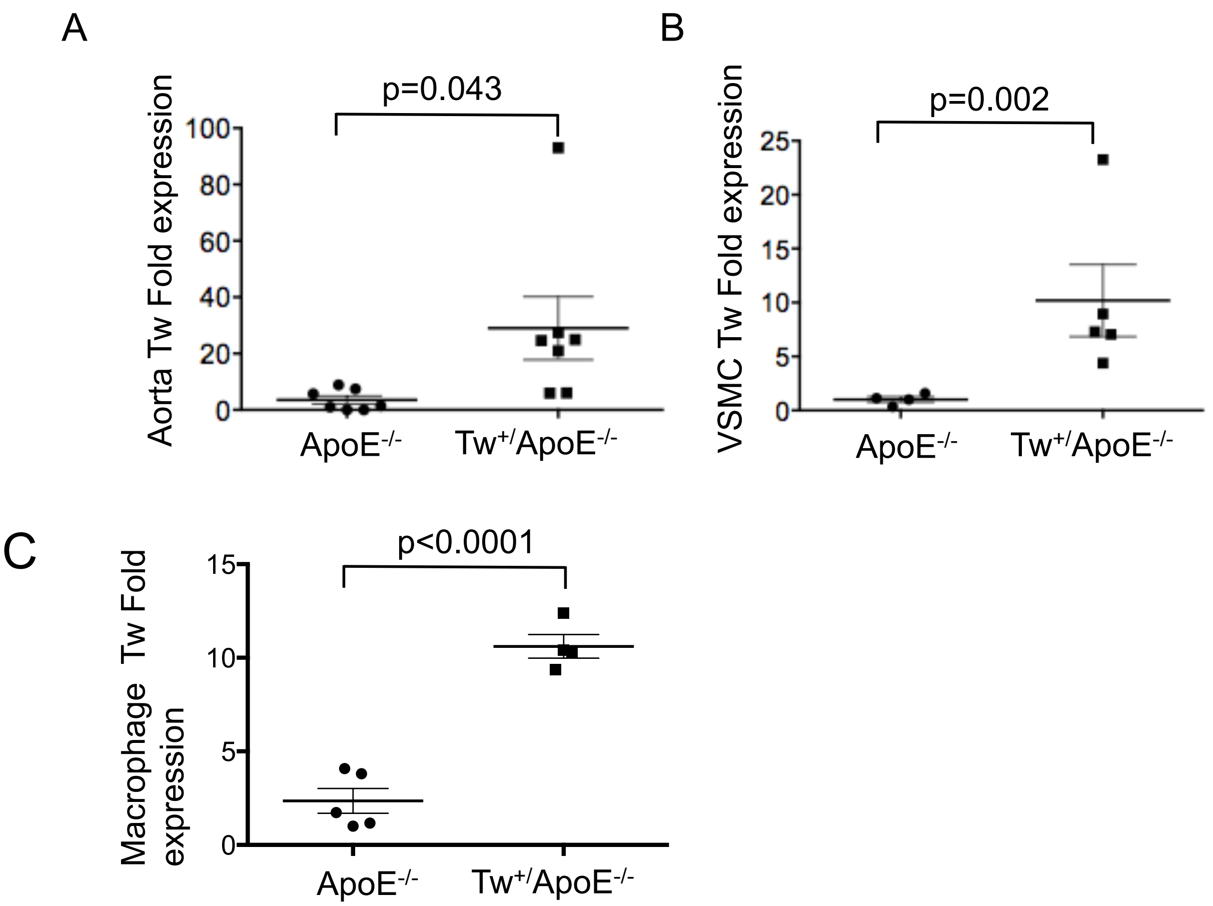

Supplemental Figure II. Twinkle mice show increased levels of twinkle expression in aortas, VSMCs and macrophages

qPCR for fold expression of Twinkle mRNA in (**A)** aortas after 14 weeks HFD, (**B**) cultured VSMCs or (**C**) cultured bone marrow-derived macrophages from Control ApoE$^{-/-}$ mice or Tw^{+}/ApoE$^{-/-}$ mice. Data are expressed as fold expression in Tw$^{+/}$ApoE$^{-/-}$ vs. ApoE$^{-/-}$ mice. n=5-7.

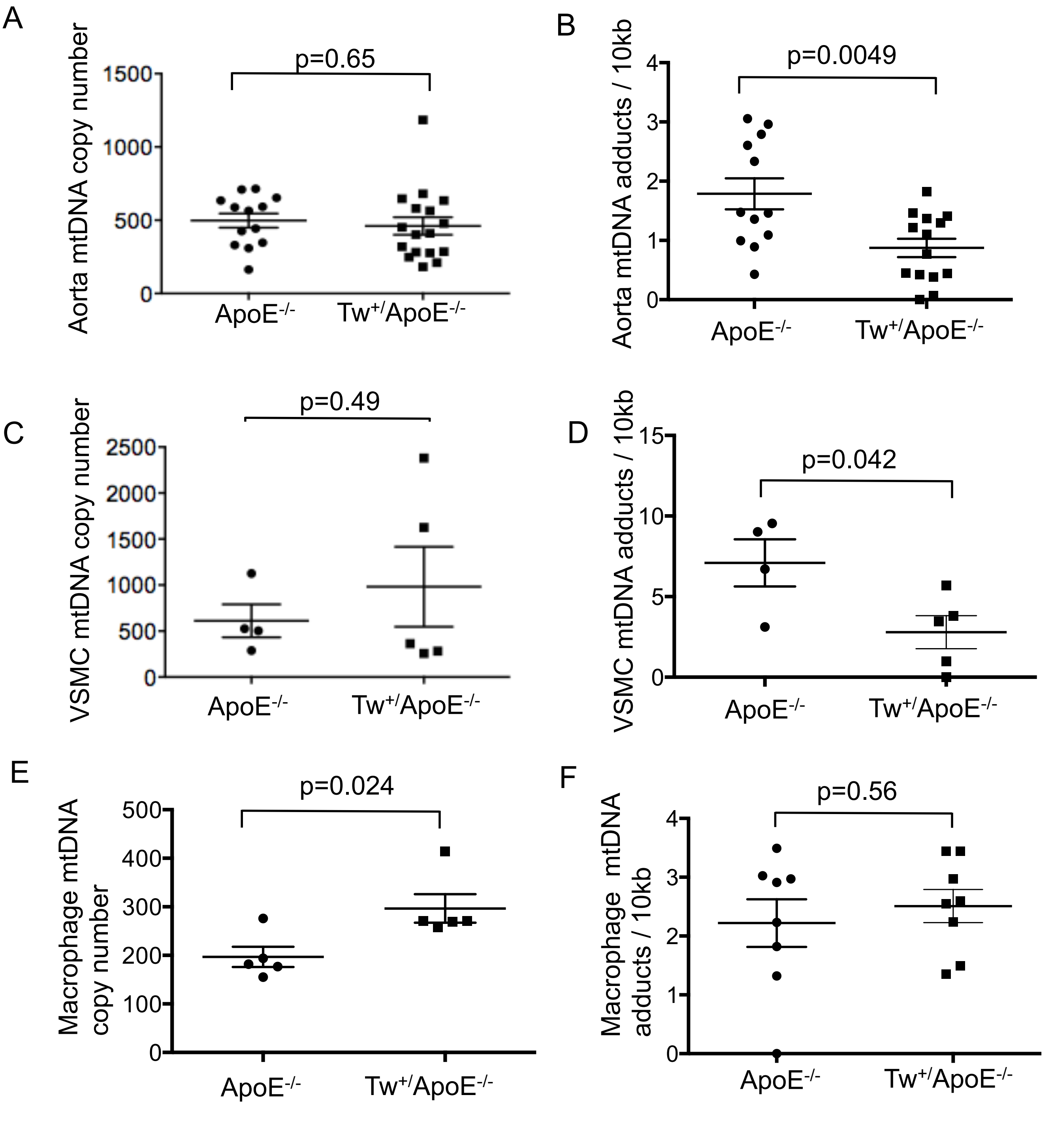

Supplemental Figure III: Twinkle mice show tissue-specific increases in mtDNA copy number or reductions in mtDNA adducts

(A-F) MtDNA copy number and mtDNA adducts in aorta **(A-B)**, VSMCs **(C-D)** or bone marrow-derived macrophages **(E-F)** of control $ApoE^{-/-}$ and $Tw^{+}/ApoE^{-/-}$ mice. (n=4-17).

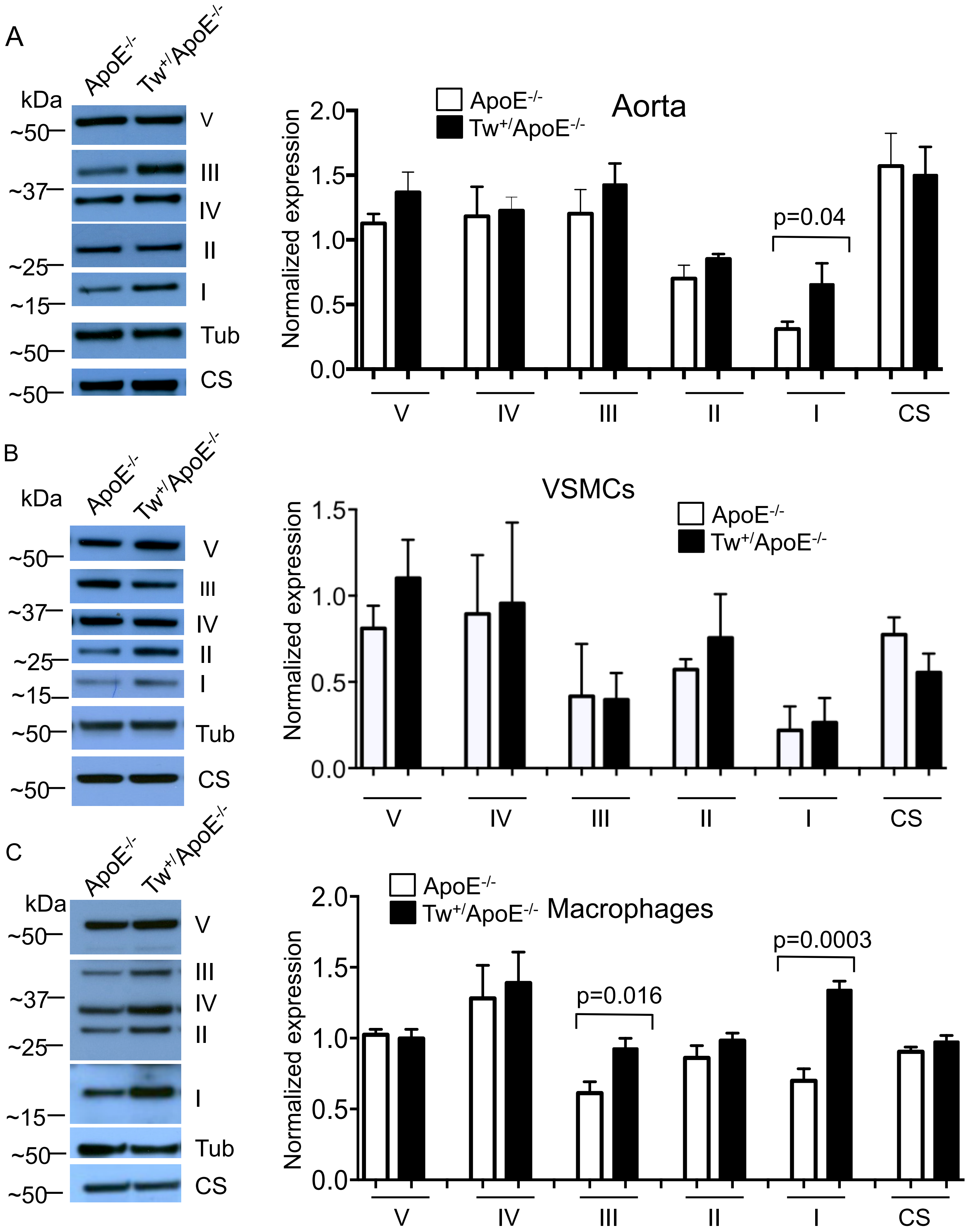

Supplemental Figure IV. Twinkle increases expression of mitochondrial respiratory complexes in aorta and macrophages

(A-C) Western blot (left) and quantification of western blots (right) for mitochondrial respiratory complex I-V or citrate synthase (CS) expression from aorta **(A)**, VSMCs **(B)**, and bone marrow-derived macrophages **(C)** derived from control ApoE$^{-/-}$ or Tw^{+}/ApoE$^{-/-}$ mice n=4-6. Tub = tubulin.

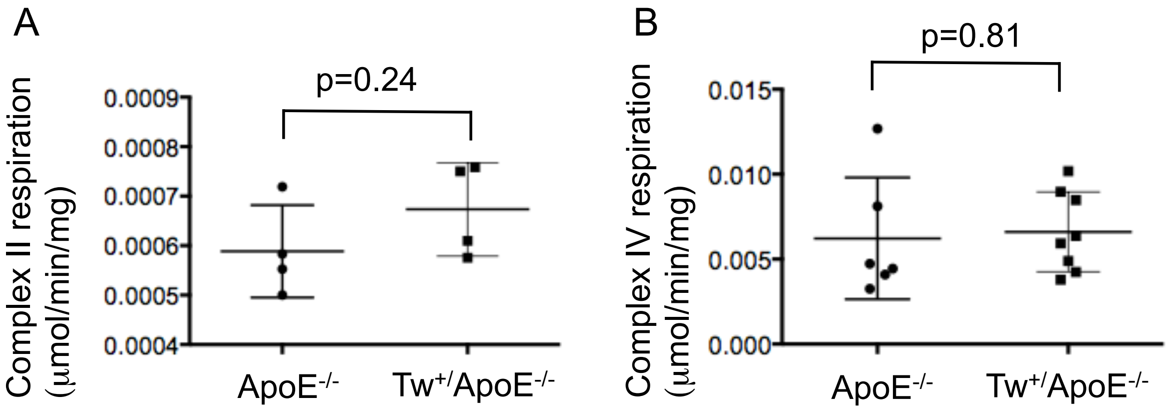

Supplemental Figure V. Twinkle mice show no difference in aortic complex II or IV respiration. Ex-vivo respirometry for complex II (**A**) and complex IV (**B**)-supported respiration in aortas from control ApoE$^{-/-}$ or Tw^{+}/ApoE$^{-/-}$ mice after 14 weeks HFD (n=4-8).

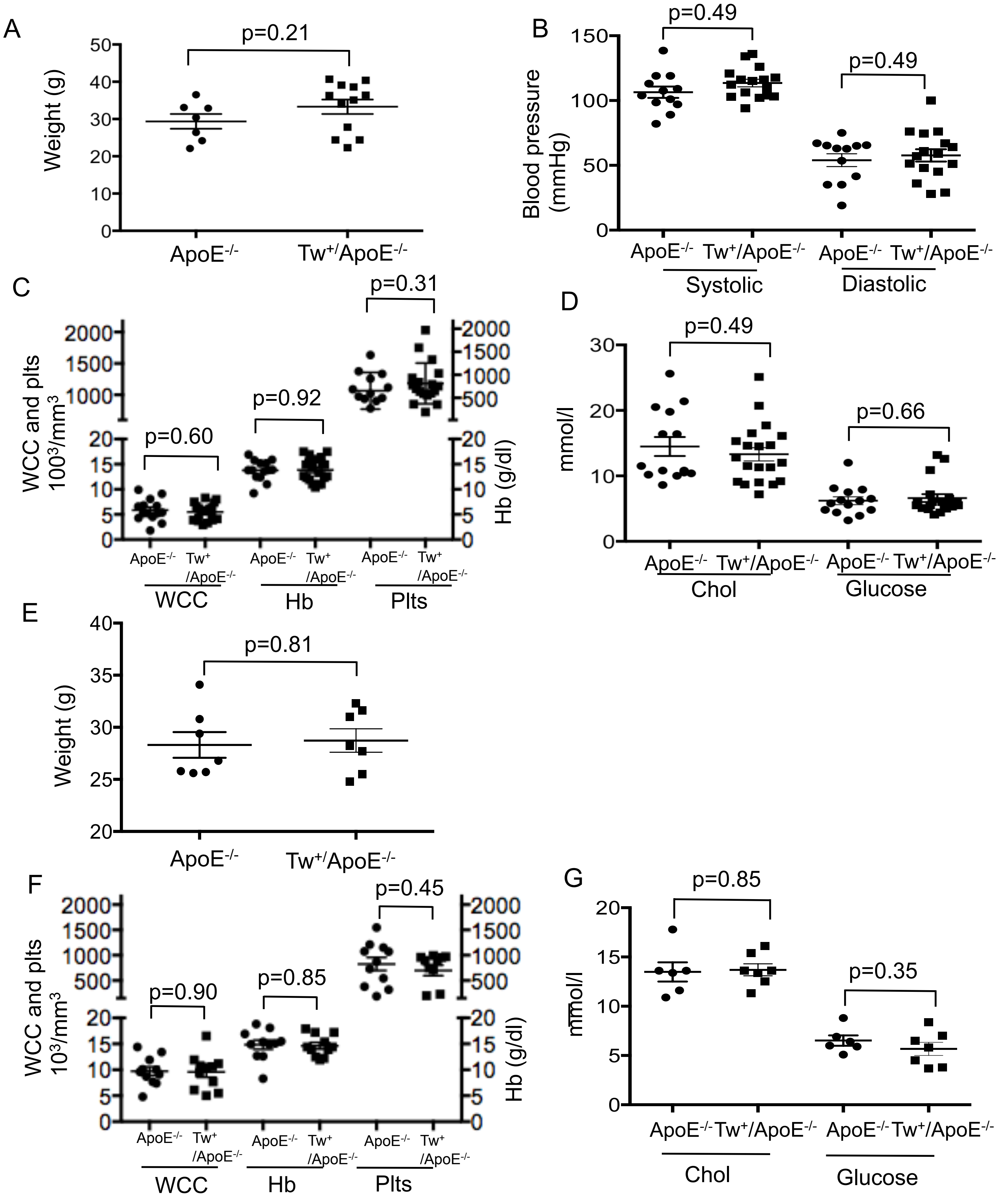

Supplemental Figure VI. Twinkle mice show normal blood counts, weights and blood pressure
Body weight, blood pressure, white cell count (WCC), Hemoglobin concentration (Hb) and platelet count (Plts), serum cholesterol (Chol) or glucose in control ApoE$^{-/-}$ and Tw^{+}/ApoE$^{-/-}$ mice **(A-D)** or BMT of ApoE$^{-/-}$ mice with control ApoE$^{-/-}$ or Tw^{+}/ApoE$^{-/-}$ marrow **(E-G)** and undergoing 14w HFD. n=7-14 **(A-D)**, and 7-11 **(E-G)**.

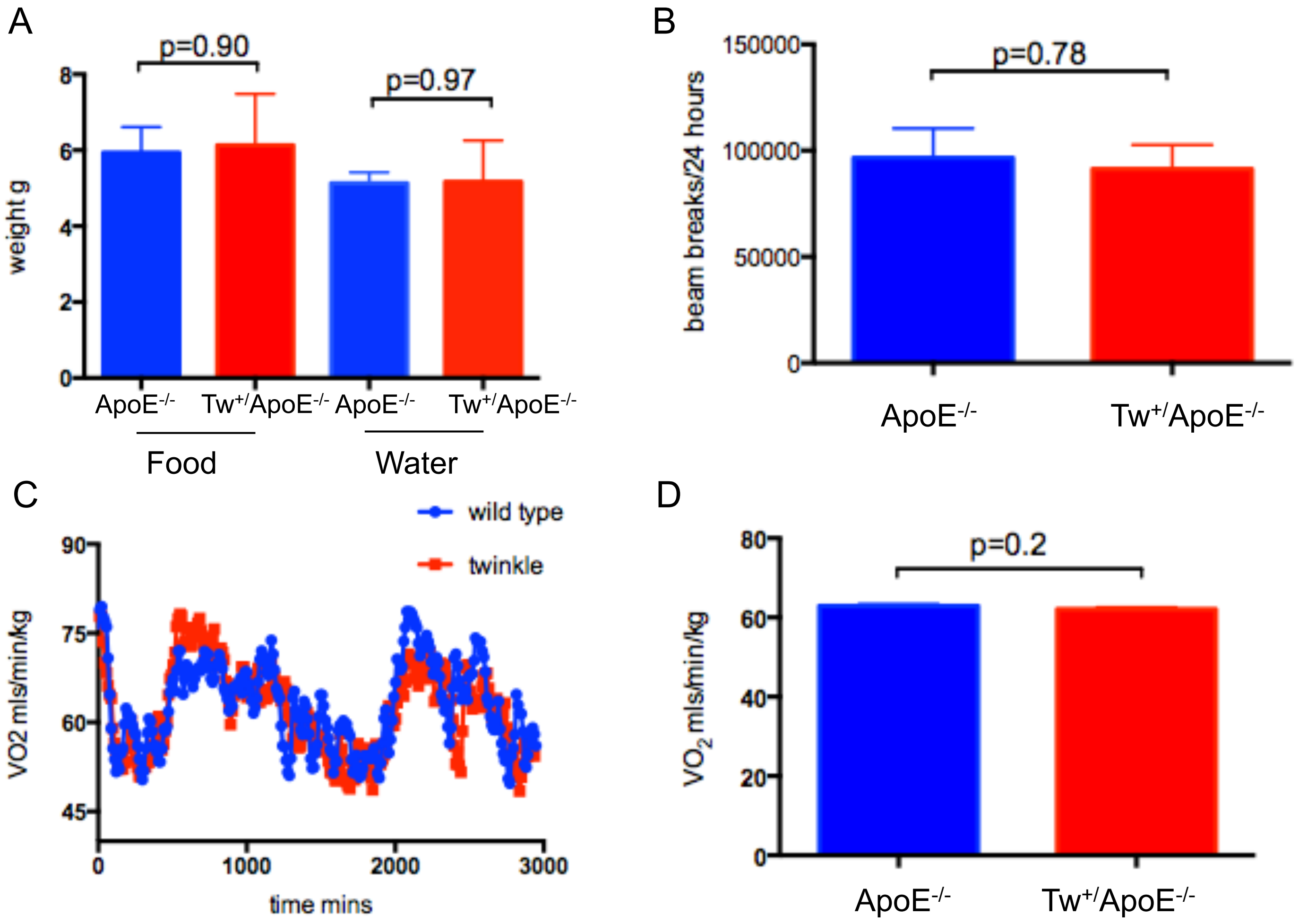

Supplemental Figure VII. Twinkle mice show normal activity and total oxygen consumption
Comprehensive laboratory animal monitoring system data of control $ApoE^{-/-}$ and $Tw^{+}/ApoE^{-/-}$ mice after 13 weeks of HFD (n=3). Food and water intake **(A).** Activity measured as the total number of beam breaks per 24 hours (**B**) . Oxygen consumption (VO_2) profile (**C**) or mean VO_2 normalised to body mass **(D**).

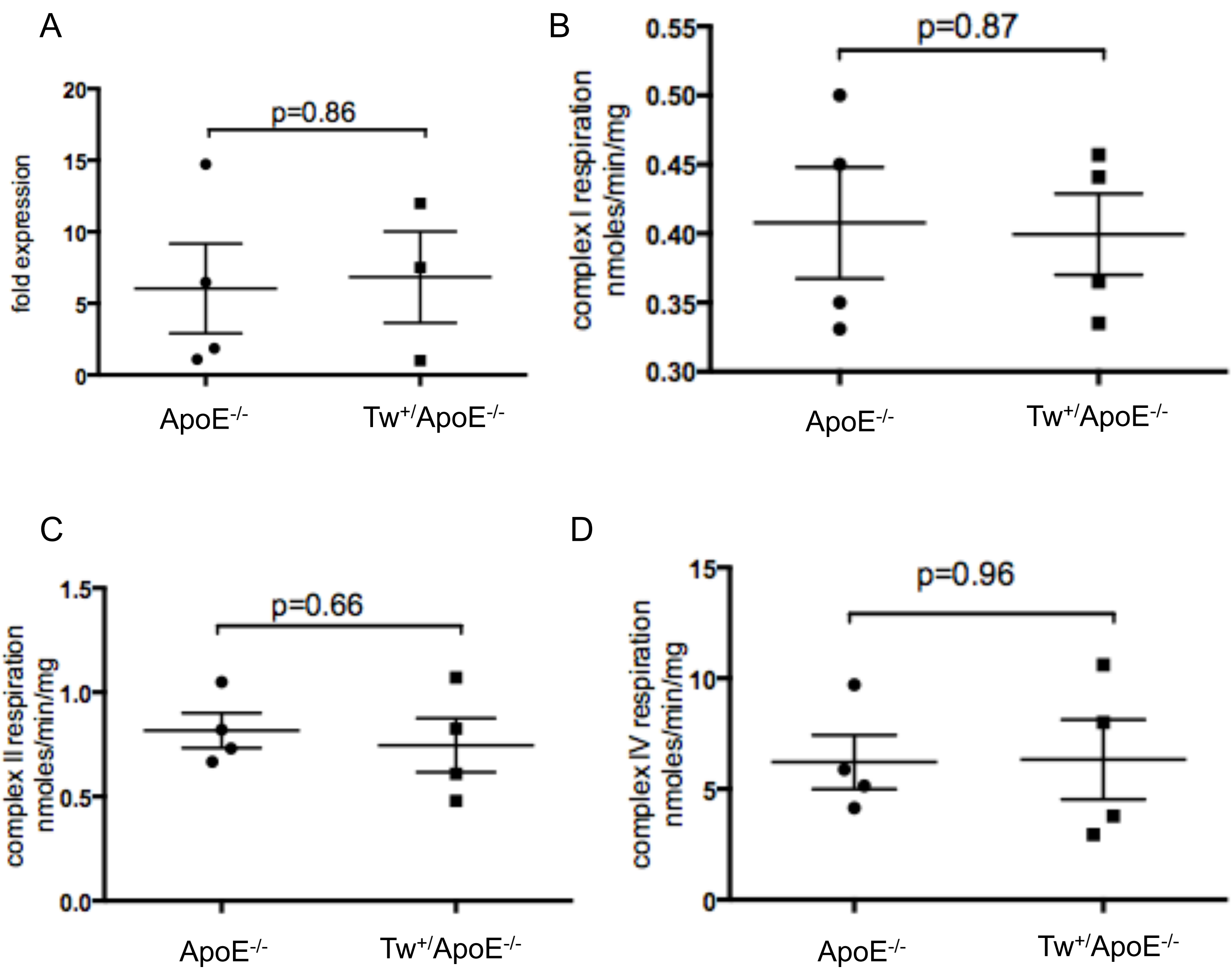

Supplemental Figure VIII. Tw+ bone marrow transplantation has no effect on aortic twinkle expression or respiration

(A-C) ApoE$^{-/-}$ mice were transplanted with control ApoE$^{-/-}$ mice or Tw^{+}/ApoE$^{-/-}$ bone marrow and fat fed from 6-20w (n=3-4) and aortas examined for **(A)** twinkle expression, Complex I (**B**), II (**C**) and IV-supported respiration (**D**). Data for Tw expression are expressed as fold expression versus ApoE$^{-/-}$ mice.

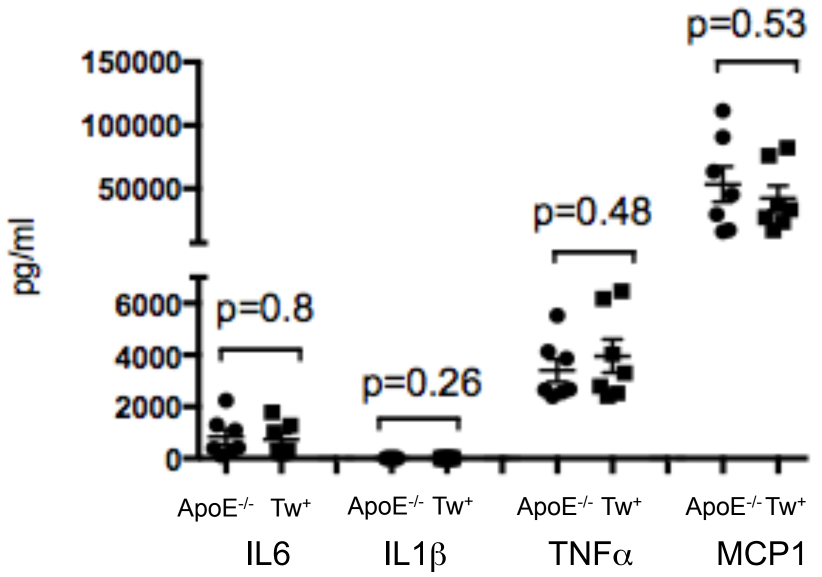

Supplemental Figure IX. Tw$^+$/ApoE$^{-/-}$ macrophages show no difference in cytokine release

Cytokine release after treatment with 1μg/ml LPS from Tw$^+$/ApoE$^{-/-}$ (Tw$^+$) and ApoE$^{-/-}$ bone marrow derived macrophages (n=7). IL indicates interleukin, TNFα, tumour necrosis factor-α and MCP1, monocyte chemoattractant protein-1.
