# Supplementary material for: Mitochondrial Respiration Is Reduced in Atherosclerosis, Promoting Necrotic Core Formation and Reducing Relative Fibrous Cap Thickness
Source: Arterioscler Thromb Vasc Biol. 2017 Sep 28;37(12):2322–32. doi: 10.1161/ATVBAHA.117.310042 (PMC5701734; doi:10.1161/ATVBAHA.117.310042)
Supplement: Supplementary file 3 [file atv-37-2322-s003.pdf]

Materials and Methods

Mice

This research has been regulated under the Animals (Scientific Procedures) Act 1986 Amendment Regulations 2012 following ethical review by the University of Cambridge Animal Welfare and Ethical Review Body (AWERB). 6-week old male and female C57Bl6/J $ApoE^{-/-}$ mice (Stock number 002052, Jackson Laboratory, Bar Harbor, Maine) were either sacrificed or fed high fat for 14 weeks (21% fat, Special Diet Services # 829100). $ApoE^{-/-}$ mice that over-expressed Twinkle ($Tw^+/ApoE^{-/-}$) were generated by crossing $C57Bl6Tw^+$ (from Dr Anu Suomalainen, Helsinki) with C57Bl6 $ApoE^{-/-}$ mice at least 3 times. Genotyping of $ApoE^{-/-}$ mice was performed by PCR using company protocols (Jackson Laboratory). Tw^+ mice genotyping was performed by PCR using the following primers at 5µmol/L: FWD: TGCAGTTCATGATGGGTCAC, REV: TGCTGTCTGCAGTTCCTTGT. The cycling parameters were 1 cycle of 94^0C, 1 min and 30 cycles of 94^0C, 30 s; 56^0C, 30s; 72^0C, 1min. Expected product sizes were Tw^+ allele 200 bp and wild type allele 350 bp.

Both male and female littermate experimental ($Tw^+/ApoE^{-/-}$) and control ($ApoE^{-/-}$) mice were fat-fed from 6-20 weeks. (21% fat, Special Diet Services # 829100). Blood pressure was measured at 16 weeks of age, using Visitech BP-2000 Blood Pressure Analysis System (Visitech Systems Inc, USA) after prior familiarisation. At 2 weeks prior to sacrifice, fasting peripheral blood samples were collected. Full blood count was assayed using the ABC vet blood counter (ABX vet 16p, ABX Diagnostics Inc, USA). Lipid profiles and glucose were assayed on serum, using commercial enzymatic assays and high-performance liquid chromatography (Department of Clinical Biochemistry, Addenbrooke's Hospital). After 13 weeks of high fat feeding, experimental mice were transferred to individual monitoring cages, kept at 22^0C, under an alternating 12h:12h light-dark cycle. Following 24 hours of acclimatisation, mice were assessed for 48 hours. Water and food consumption, ambulatory activity, VCO_2 and VO_2 were measured in the comprehensive laboratory animal monitoring system (CLAMS, Columbus Instruments, OH, USA) fitted with an indirect calorimetry system (miniMOX; University of Cambridge, Cambridge, UK). Body weight was assessed before and after the procedure, and VO_2 corrected for lean body weight.

For bone marrow transplantation experiments, 6-week old $ApoE^{-/-}$ mice received 2 doses of 5.5Gy total body irradiation, 4 hours apart. For repopulation, bone marrow cells were harvested from either $Tw^+/ApoE^{-/-}$ or $ApoE^{-/-}$ mice. Tibias and fibulas were excised from donors, and after removal of the trabecular heads, the bone marrow was flushed out with Dulbecco's Modified Eagle Medium (DMEM). The cells were washed with phosphate-buffered saline (PBS) and $10x10^6$ cells were injected via the tail vein, 6 hours after the 1st dose of irradiation. Recipient mice received 0.025% Baytril antibiotic (Bayer AG, Germany) in their drinking water for 4 weeks, and peripheral counts were performed at 11 and 18 weeks of age to check for reconstitution. At sacrifice the mice underwent terminal anaesthesia with pentobarbital, and blood and tissues were removed.

In vivo mitochondrial hydrogen peroxide assessment

MitoB, a mitochondria-targeted ratiometric probe, was used to assess *in vivo* mitochondrial H_2O_2. MitoB has a triphenylphosphonium cation, which drives its accumulation within mitochondria, where it reacts with H_2O_2 to form MitoP. Quantifying the mitoP/mitoB ratio enables measurement of mitochondrial H_2O_2. On the day of sacrifice, mice were administered 25 nmol of mitoB via intravenous injection. After 4 hours of equilibration, tissues were harvested for analysis of mitoP/mitoB by liquid chromatography-tandem mass spectrometry[1].

Histological analysis

Ascending aortas and brachiocephalic arteries were fixed in 10% formalin, embedded in paraffin and sectioned at 5 µm intervals. The specimens were then stained with Masson's Trichrome. Images were captured and analysed in Image-Pro Insight (Media Cybernetics) to

assess atherosclerotic lesion size and composition. Total plaque area was expressed as a percentage of vessel lumen area, and fibrous cap and necrotic core areas expressed as a percentage of plaque area. Descending aortas were dissected and incised longitudinally. The samples were washed with de-ionised water and 60% isopropranolol, before staining with 60% Oil Red O stain. Images were captured and the stained area was expressed as a percentage of total vessel area[2].

For immunohistochemical analysis of aortic root plaques, sections were blocked in bovine serum albumin (BSA) and incubated with primary antibodies specific for anti-smooth muscle actin (SMA, 1A4, Dako,1:500) and Ki67 (VP-K452-Ki67, Vector Laboratories,1:100). Sections were stained with biotinylated secondary antibodies detected with Avidin/Biotinylated enzyme Complex (ABC) reagents (Vector Laboratories), visualised with Vector Blue or diaminobenzidene (DAB, Vector Laboratories). Terminal deoxynucleotidyl transferase deoxyuridine triphosphate (dUTP) Nick End Labelling (TUNEL) assay was used for the detection of apoptosis. Slides were incubated with terminal deoxynucleotidyl transferase (TdT) and digoxigenin-dUTP (Roche). Detection was performed with an alkaline phosphatase-conjugated antibody to digoxigenin (Roche), visualised with 5-bromo-4-chloro-3-indoyl-phosphate/p-nitroblue tetrazolium (Vector). 1% eosin was used as the counterstain. Digital images were acquired, and stained areas were expressed as a percentage of total plaque area (for SMA), or the number of positive cells was expressed as a percentage of the total number of plaque cells (Ki67 and TUNEL staining)[3].

Human atherosclerosis
Following Ethical Committee approval (REC97/084), human atherosclerotic plaques were obtained following carotid endarterectomy, and normal aortas from patients undergoing aortic valve surgery or root replacement. DNA was isolated for mtDNA copy number from whole plaques or normal aorta. Plaques were also microdissected into regions containing the fibrous cap, shoulder region, necrotic core, and underlying media for analysis by Seahorse (see below). Human VSMCs were cultured from normal aortas and plaques as previously described[4] and used below passage 3 for Seahorse, or expanded for lentivirus infection, qPCR or Western blotting.

Seahorse assay
Mitochondrial respiration was determined using a Seahorse XF24e extracellular flux analyzer (Seahorse Bioscience) as per the manufacturer's instructions. Five tissue pieces of approximately 1 mg were dissected from carotid endarterectomies into regions representing underlying media, shoulder region, cap and core and placed into 24-well Seahorse islet capture plates filled with 250 µl pre-warmed Dulbecco's modified Eagle's medium (DMEM) sparing four temperature control wells. Islet capture screens were applied, medium replaced with 250 µl fresh DMEM and remaining air bubbles removed. Final assay concentrations were: oligomycin 10 µg/ml, FCCP 1 µmol/L, rotenone 10 µmol/L and antimycin 10 µmol/L as described previously[5]. Four measurements were obtained at baseline and following injection of the above compounds. Following analysis, all tissue pieces were retrieved, excess fluid removed, weighed and fixed in neutral-buffered formalin before staining with hematoxylin and eosin (H+E) for confirmation of segment identity and assessment of cell number.

For cells, VSMCs or macrophages were plated into XF96 microplates and allowed to adhere for 24 hours. Basal oxygen consumption rate (OCR) was determined, and then sequential additions of oligomycin (1 µg/ml), FCCP (12 µmol/L) and antimycin/rotenone (10/1 µmol/L) performed. OCR was normalized to either wet weight and cell nuclei (plaque tissue) or protein content (cells) determined by the Bradford method[6].

Quantitative PCR for mtDNA damage and copy number

To assess for global mtDNA damage, amplification of a 10 kb segment of mitochondrial DNA spanning 60% of the mitochondrial genome was performed. To control for mtDNA copy number, this was compared to the amplification of a short target.

Long primers: used at 0.5 μM producing a 10150 bp product: FWD: GCCAGCCTGACCCATAGCCATAAT, REV: GAGAGATTTTATGGGTGTAATGCG. Short primers: used at 0.5 μM producing a 127 bp product: FWD: GCCAGCCTGACCCATAGCCATAAT, REV: GCCGGCTGCGTATTCTACGTTA.

QPCR was performed in triplicate, on 20 ng of sample DNA, in 20 μl reactions on a Rotor-Gene™ 6000 QPCR thermocycler (Qiagen). Rotor-gene SYBR Green Mix (Qiagen) was used for the short reaction. PFU Ultra II Hotstart PCR Master Mix (#600850 Agilent) was used for the long reaction, with 1 in 400 dilution of Eva Green (#31000 Biotium). Cycling parameters for the short reaction were 95ºC for 5 minutes followed by 45 cycles of 95ºC for 5s, 60ºC for 10s. Conditions for the long amplification were 94ºC for 2 minutes followed by 45 cycles of 92ºC for 30s, 71ºC for 30s, and 68ºC for 5 minutes. Ct values were identified using the Rotorgene software, and the relative amplification of the 10kb product calculated by the comparative Ct method[7]. DNA lesion frequencies were calculated using the Poisson transformation[8].

For assessment of mouse mitochondrial DNA copy number, the amplification of a short segment of mtDNA was compared to the amplification of a short segment of nuclear (β globin) DNA[9]. The short mtDNA primers were used as above; Primers used at 0.5 μmol/L were as follows: MtDNA primers: producing a 127 bp product,
FWD: GCCAGCCTGACCCATAGCCATAAT
REV: GCCGGCTGCGTATTCTACGTTA.

Nuclear primers: producing a 155 bp product
FWD: TTGAGACTGTGATTGGCAATGCCT,
REV: CCAGAAATGCTGGGCGCTCACT.

Human mitochondrial DNA copy number was assessed using primers (900nmol/L) that have previously been described[10]. Relative mtDNA expression was calculated for two short segments and the average of these measurements was used.

Mitochondrial primers:
FWD: CACCCAAGAACAGGGTTTGT
REV: TGGCCATGGGTATGTTGTTAA

FWD: CGTCATTATTGGCTCAAC
REV: GATGGAGACATACAGAAATAG

Nuclear primers:
FWD: TGCTGTCTCCATGTTTGATGTATCT
REV: TCTCTGCTCCCCACCTCTAAGT

QPCR was performed in triplicate, on 10 (human) -20 (mouse) ng of sample DNA, on a Rotor-Gene™ 6000 QPCR thermocycler (Corbett Research). Rotor-gene SYBR Green Mix (Qiagen) was used for the reaction, with the following cycling parameters: Mouse: 95ºC for 5 minutes followed by 45 cycles of 95ºC for 5s, 60ºC for 10s. Human: 50ºC for 2 minutes and 95ºC for 10 minutes followed by 40 cycles of 95ºC for 15s and 60ºC for 60s. Relative expression was calculated by the comparative Ct method[7].

RNA isolation and Quantitative PCR

As per manufacturer's instructions, RNA was isolated from aortas and cells using TRIzol Reagent (Thermo Fisher Scientific). RNA was treated with RQ1 RNase-Free DNase treatment and cDNAs synthesized using Oligo(dT) primers and reverse transcription system (Promega). QPCR analysis for was performed using the following primers at 0.5 µmol/L:

Gene	Forward	Reverse
Twinkle	GCCACGTGACTCTGGTCATTC	CCATCAAAGCGATTCTTGGACA
β actin	GGCACCACACCTTCTACAATG	GTGGTGGTGAAGCTGTAGCC

QPCR was performed in triplicate, in 20 µl reactions. Reaction reagents and conditions were as described for copy number qPCR.

Western blotting
Western blotting was performed on 20 µg of aortic, plaque or cellular protein loaded on to a 4-12% polyacrylamide gel and separated by electrophoresis. Proteins were transferred to PVDF membranes, blocked for 1 h in 5% non-fat milk and probed. Primary antibodies included total OXPHOS rodent antibody (1:500)(ab110413), citrate synthase (1:500-1:1000)(ab96600), manganese superoxide dismutase (1:5000)(ab13533), DNA polymerase gamma (1:1000)(ab128899), actin (1:10000)(ab8227)(all from abcam), tubulin (1:500)(2148s), PINK1, (1:1000)(6946), TFAM (1:1000)(7495s)(all from Cell Signaling Technologies), and PEO1 (Twinkle)(1:1000)(ARP36483_P050)(from Aviva Systems Biology).

Aortic respiration
To assess mitochondrial respiration, descending aortas were dissected and maintained in preservation solution (BIOPS: 10mM Ca-EGTA buffer, 0.1 µM free calcium, 20 mM imidazole, 20 mM taurine, 50 mM MES, 0.5 mM dithiothreitol, 6.56 mM $MgCl_2$, 5.77 mM ATP, 15 mM phosphocreatine, pH 7.1) at 4°C. Samples were permeabilized in 50 µg/ml saponin solution and washed in respiration buffer (0.5 mM EGTA, 3 mM $MgCl_2 \cdot 6H_2O$, 60 mM K-lactobionate, 20 mM taurine, 10 mM KH_2PO_4, 20 mM HEPES, 110 mM mannitol, 0.3 mM dithiothreitol, 1 mg/ml fatty acid free BSA, pH 7.1). Respiration was assessed at 37°C with a Clarke-type oxygen electrode (Strathkelvin Instruments Ltd, UK).

Complex I-supported respiration rates were acquired using 10 mM glutamate and 5 mM malate. 5mM ADP was then added to stimulate State 3 respiration. After the addition of 1 µM rotenone, complex II-supported respiration was assessed with 10 mM succinate. Following complex III inhibition by 5 µM antimycin, 0.5 mM *N,N,N',N'*-Tetramethyl-*p*-phenylenediamine (TMPD) and 2 mM ascorbate were used to induce complex IV respiration. The intactness of the outer mitochondrial membrane was assessed by adding 10 µM cytochrome C**.** Tissues were removed from the electrode chambers and dried, with oxygen flux expressed as nanomoles O_2 per minute per dry weight[11]**.**

Cell isolation and culture
For the differentiation of bone marrow-derived macrophages (BMDMs) bone marrow cells were isolated and cultured for 7 days in RPMI 1640 (Invitrogen) supplemented with 20% Fetal Bovine Serum (FBS) and 15% L929-conditioned medium[12]. Isolation purity was determined by flow cytometric staining for CD11b and F4/80. Vascular smooth muscle cells (VSMCs) were cultured from explanted aortas. The aortas were dissected free of surrounding fat and connective tissue, divided into small pieces, and cultured with Dulbecco's Modified Eagle Medium (DMEM) (Sigma UK) supplemented with 20% FBS.

Cellular ROS assay

To assess ROS cells were incubated in 10 µM 5-(and 6) 2'7'dichlorodihydrofluorescein diacetate (DCFDA) (Invitrogen #C6827) for 30 minutes at 37^0C. Cells were washed with PBS and relative fluorescence index was measured (Excitation 485 nm, Emission 528 nm).

Cellular proliferation and apoptosis assays
VSMC proliferation was measured using the CyQuant cell assay (Invitrogen), a nucleic acid-based fluorescent assay, according to the manufacturer's instructions. Fluorescence was measured on a Synergy HT Plate Reader (Biotek Instruments) with standard curves generated from known numbers of cells. For macrophage and VSMC apoptosis assessment, cells were seeded into 24-well plates and incubated with 50 µmol/L tert-butyl hydroperoxide (t-BHP) for 16 hours. Cells were then stained with propidium iodide and Annexin V Alexa Fluor 488 (Thermo Fisher Scientific) as per manufacturer's instructions, and analyzed on an Accuri C6 flow cytometer (Becton Dickinson).

Cytokine assay
Bone marrow derived macrophages were incubated with 1 µg/ml lipopolysaccharide (LPS) *E. coli* strain 0111:B4 (Sigma # L2630) for 16 hours. The culture supernatant was collected and analysed for cytokines using Invitrogen mouse bead assays (Invitrogen) on a Becton Dickinson Accuri C6 flow cytometer (Becton Dickinson Inc, USA). Known concentrations of standards were used to generate standard curves, and cytokine concentrations calculated using FlowCytomix Pro software (eBioscience, USA).

Mitophagy assay
The plasmid containing mitochondrially-targeted Keima (Keima-pLESIP) was a gift from Dr. Toren Finkel, NHLBI, USA. Plasmids were propagated in One Shot OmniMAX™ 2-T1R chemically-competent *E. coli*. HEK-293T cells were co-transfected with packaging plasmids pMD2.G, psPAX and Keima-pLESIP using FuGENE 6 (Roche) according to standard techniques. The transfection medium was replaced with fresh culture medium after 24 hours, pseudo-lentivirus particles were harvested after 48 hours, concentrated using Amicon Ultra-15 centrifugal filter devices and lentivirus titers determined using qPCR lentivirus titration (titer) kit (abm, LV900) according to manufacturer's instructions. VSMCs were transduced at an MOI of three in the presence of 8 µg/ml Polybrene (Sigma) and after an overnight incubation the medium was replaced with fresh culture medium. Infected cells were puromycin-selected and plated into MatTek gamma irradiated, poly-d-lysine coated, 35mm glass bottom wells before treatment, co-staining with Hoechst 33342 and imaging using an SP5 confocal microscope (Leica).

Assessment of mitophagy using mitochondrial keima has previously been described[13]. In short, fluorescence of keima was imaged in two separate channels after consecutive excitation at 458nm (coded green) and 561nm (coded red) using a 580nm to 690nm emission range. Hoechst was imaged in a third channel after excitation by UV light. At least 20 z-stacks per group were acquired in at least three independent experiments from three different primary cell cultures per group using a 63x/1.40NA oil immersion objective. ImageJ plug-in, originally designed for assessing mitochondrial morphology (Richard Butler, Gurdon Inst, Cambridge), was used to quantify fluorescence and the mitophagy index (ratio red/green) as a marker of mitophagy was calculated.

Statistical analysis
Data were tested for a normal distribution by examination of the histogram and normal probability plot, and by using the Shapiro-Wilk test. Student's t test was used for pair-wise comparison for data following a normal distribution, and Mann Whitney U test for non-parametric data. ANOVA with Bonferroni's correction was used to compare means of multiple groups. Values are presented as means ± SEM. $p<0.05$ was considered significant.

References

1. Cocheme HM, Logan A, Prime TA, Abakumova I, Quin C, McQuaker SJ, Patel JV, Fearnley IM, James AM, Porteous CM, Smith RA, Hartley RC, Partridge L, Murphy MP. Using the mitochondria-targeted ratiometric mass spectrometry probe MitoB to measure H2O2 in living Drosophila. *Nat Protoc.* 2012;7:946-958.
2. Mercer J, Figg N, Stoneman V, Braganza D, Bennett MR. Endogenous p53 protects vascular smooth muscle cells from apoptosis and reduces atherosclerosis in ApoE knockout mice. *Circ Res.* 2005;96:667-674.
3. Mercer JR, Gray K, Figg N, Kumar S, Bennett MR. The Methyl Xanthine Caffeine Inhibits DNA Damage Signaling and Reactive Species and Reduces Atherosclerosis in $ApoE^{-/-}$ Mice. *Arterioscler Thromb Vasc Biol.* 2012;32:2461-2467.
4. Gorenne I, Kumar S, Gray K, Figg N, Yu H, Mercer J, Bennett M. Vascular smooth muscle cell sirtuin 1 protects against DNA damage and inhibits atherosclerosis. *Circulation.* 2013;127:386-396.
5. Feeley K, Westbrook D, Bray A, Ballinger S. An ex-vivo model for evaluating bioenergetics in aortic rings. *Redox Biol.* 2014;2:1003-1007.
6. Bradford M. A rapid and sensitive method for the quantitation of microgram quantities of protein utilizing the principle of protein-dye binding. *Anal Biochem.* 1976;72:248-254.
7. Schmittgen TD, Livak KJ. Analyzing real-time PCR data by the comparative C(T) method. *Nat Protoc.* 2008;3:1101-1108.
8. Yakes FM, Van Houten B. Mitochondrial DNA damage is more extensive and persists longer than nuclear DNA damage in human cells following oxidative stress. *Proc Natl Acad Sci USA.* 1997;94:514-519.
9. Guo W, Jiang L, Bhasin S, Khan S, Swerdlow R. DNA extraction procedures meaningfully influence qPCR-based mtDNA copy number determination. *Mitochondrion.* 2009;9:261-265.
10. Gammage P. *Engineered mtZFNs for Manipulation of Human Mitochondrial DNA Heteroplasmy*. Berlin:: Springer; 2016.
11. Yu E, Calvert PA, Mercer JR, Harrison J, Baker L, Figg NL, Kumar S, Wang JC, Hurst LA, Obaid DR, Logan A, West NE, Clarke MC, Vidal-Puig A, Murphy MP, Bennett MR. Mitochondrial DNA damage can promote atherosclerosis independently of reactive oxygen species through effects on smooth muscle cells and monocytes and correlates with higher-risk plaques in humans. *Circulation.* 2013;128:702-712.
12. Weischenfeldt J, Porse B. Bone Marrow-Derived Macrophages (BMM): Isolation and Applications. *CSH Protoc.* 2008:prot5080.
13. Sun N, Yun J, Liu J, Malide D, Liu C, Rovira, II, Holmstrom KM, Fergusson MM, Yoo YH, Combs CA, Finkel T. Measuring In Vivo Mitophagy. *Mol Cell.* 2015;60:685-696.
